# Fecal Volatile Organic Compound Profiles are Not Influenced by Gestational Age and Mode of Delivery: A Longitudinal Multicenter Cohort Study

**DOI:** 10.3390/bios10050050

**Published:** 2020-05-11

**Authors:** Nancy Deianova, Sofia el Manouni el Hassani, Hendrik J. Niemarkt, Veerle Cossey, Anton H. van Kaam, Floor Jenken, Mirjam M. van Weissenbruch, Esmee M. Doedes, Kyra Baelde, Renee Menezes, Marc A. Benninga, Wouter J. de Jonge, Nanne K. de Boer, Tim G. de Meij

**Affiliations:** 1 Department of Pediatric Gastroenterology, Amsterdam UMC, Academic Medical Center, 1105 AZ Amsterdam, The Netherlands; s.elmanounielhassani@amsterdamumc.nl (S.e.M.e.H.); m.a.benninga@amsterdamumc.nl (M.A.B.); t.demeij@amsterdamumc.nl (T.G.d.M.); 2 Department of Pediatric Gastroenterology, Amsterdam UMC, VU University Medical Center, 1081 HV Amsterdam, The Netherlands; e.doedes@amsterdamumc.nl (E.M.D.); k.baelde@amsterdamumc.nl (K.B.); 3 Neonatal Intensive Care Unit, Máxima Medical Center, 5504 DB Veldhoven, The Netherlands; Hendrik.Niemarkt@mmc.nl; 4 Neonatal Intensive Care Unit, University Hospitals Leuven, 3000 Leuven, Belgium; veerle.cossey@uzleuven.be; 5 Neonatal Intensive Care Unit, Amsterdam UMC, VU University Medical Center, 1081 HV Amsterdam, The Netherlands; a.h.vankaam@amsterdamumc.nl (A.H.v.K.); m.vanweissenbruch@amsterdamumc.nl (M.M.v.W.); 6 Neonatal Intensive Care Unit, Amsterdam UMC, Academic Medical Center, 1105 AZ Amsterdam, The Netherlands; 7 Neonatal Intensive Care Unit, Wilhelmina Children’s Hospital/University Medical Center Utrecht, 3508 AB Utrecht, The Netherlands; fjenken@umcutrecht.nl; 8 Biostatistics Unit, Netherlands Cancer Institute (NKI), 1066 CX Amsterdam, The Netherlands; r.menezes@nki.nl; 9 Amsterdam University Medical Centers, University of Amsterdam, Tytgat Institute for Liver and Intestinal Research, Amsterdam Gastroenterology & Metabolism, 1105 BK Amsterdam, The Netherlands; w.j.dejonge@amsterdamumc.nl; 10 Department of Gastroenterology and Hepatology, Amsterdam Gastroenterology and Metabolism Research Institute, Amsterdam UMC, VU University Medical Center, 1081 HV Amsterdam, The Netherlands; khn.deboer@amsterdamumc.nl

**Keywords:** gestational age, mode of delivery, preterm infants, electronic nose, eNose, flatography, VOC, volatile organic compound, metabolomics

## Abstract

Fecal volatile organic compounds (VOC) reflect human and gut microbiota metabolic pathways and their interaction. VOC behold potential as non-invasive preclinical diagnostic biomarkers in various diseases, e.g., necrotizing enterocolitis and late onset sepsis. There is a need for standardization and assessment of the influence of clinical and environmental factors on the VOC outcome before this technique can be applied in clinical practice. The aim of this study was to investigate the influence of gestational age (GA) and mode of delivery on the fecal VOC pattern in preterm infants born below 30 weeks of gestation. Longitudinal fecal samples, collected on days 7, 14, and 21 postnatally, were analyzed by an electronic nose device (Cyranose 320^®^). In total, 58 preterm infants were included (29 infants born at GA 24–26 weeks vs. 29 at 27–29 completed weeks, 24 vaginally born vs. 34 via C-section). No differences were identified at any predefined time point in terms of GA and delivery mode (*p* > 0.05). We, therefore, concluded that correction for these factors in this population is not warranted when performing fecal VOC analysis in the first three weeks of life.

## 1. Introduction

In recent decades, new diagnostic methods and therapeutic options in the care for preterm infants have resulted in improved outcomes, particularly after extremely preterm birth [1]. Nevertheless, mortality and morbidity are still high, especially in very and extremely preterm born children [2,3,4]. Late onset sepsis (LOS) and necrotizing enterocolitis (NEC), for example, are major causes of death in this population with incidence of ca. 20% and 8% and mortality rate of more than 20% and 30%, respectively [5,6,7,8,9,10,11].

Timely detection and prompt treatment are often impeded by lack of early and specific clinical signs and diagnostic tools [12,13]. Moreover, the currently available techniques include painful, invasive venous and lumbar puncture in the LOS and/or NEC diagnostic workup [4,14]. Therefore, the development of noninvasive and early diagnostic tools remains crucial for optimization of care [13,15].

In the past decade, metabolic research has pointed to new potential biomarkers for various medical conditions, including oncologic, inflammatory, endocrine, and infectious diseases in both adults and children [16,17,18,19,20,21,22,23,24,25,26,27,28,29,30,31,32]. The sensitivity of a metabolomics approach to detect subtle alterations in metabolic pathways can, in addition, provide insight into mechanisms underlying various (patho)physiological conditions [33]. The main technologies used in identifying metabolites are untargeted and targeted mass spectrometry (MS), often used in combination with either liquid or gas chromatography (LC-MS and GC-MS, resp.) [33]. In NEC, for example, several volatile organic compounds (VOC), including (Z-)hept-2-enal, pent-1-en-3-one, 2-ethylfuran, pentanal and 2-penthylfuran, as measured by GC-MS, have been reported to predict the disease with a moderate accuracy 3–4 days prior to clinical diagnosis [27]. Although crucial for gaining (patho)physiological insight, these techniques are costly in time and resources, complex to use, and labor-intensive, which makes them less fit for bedside application [34]. Alternatively, instruments such as electronic nose (eNose) devices and field asymmetric ion mobility spectrometry (FAIMS) can provide quicker analysis of VOC based on pattern recognition [35,36].

With the latter technology, the presented VOC are first ionized and subsequently transported toward a build-in sensor using a carrier gas. During the transport, the electric field is modulated, which makes the ions drift in a ‘zigzag-like’ pattern before reaching the sensor. As a result, a wide variety of different ionized molecules can be separated by (ion-specific) differences in mobility [35].

In eNose devices, on the other hand, the odor sample is drawn across a sensor array, which results in a competitive interaction between the sensors and VOC upon exposure. Subsequently, reversible physical and/or chemical alterations in the sensing material occur, changing the electrical properties in each sensor. These changes are registered and result in a scent pattern [37].

Specifically in neonatal care, eNoses have proven their potential in diagnosis of LOS, NEC, and bronchopulmonary dysplasia (BPD) in the clinical prodrome [38,39,40,41]. To optimize their diagnostic accuracy, however, it is important to assess and correct for physiological conditions influencing the VOC composition.

Since part of excreted VOC are products of metabolic pathways of (commensal) micro-organisms, it is hypothesized that VOC in newborns would be affected by factors affecting the microbiota composition, such as mode of delivery, gestational age (GA), and feeding [42,43,44,45,46,47]. In a previous study, it was demonstrated that VOC patterns, as measured by an eNose device (Cyranose320^®^), are influenced by enteral feeding practice in preterm neonates born at GA < 30 weeks [48]. The current study focused on GA and mode of delivery. Together with other studies on the impact of pre-analytical and post-analytical variables on VOC composition, this research could contribute to methodological guidelines for future VOC research [44,45,49].

## 2. Materials and Methods

### 2.1. Subjects

The current study was part of an ongoing prospective multicenter cohort study in nine participating neonatal intensive care units (NICUs) in the Netherlands and Belgium in which infants born before 30 weeks of gestation are included [39]. The aim of that study is to identify novel noninvasive biomarkers for LOS and NEC. Of all included infants, clinical data and a fecal sample was collected daily from birth up to 28 days postnatally.

For the current study, infants born at four out of nine centers were included in order to limit center-specific variation in fecal VOC outcome: Emma Children’s Hospital (location Academic Medical Center, Amsterdam, The Netherlands), Máxima Medical Center (Veldhoven, The Netherlands), Wilhelmina Children’s Hospital (Utrecht, The Netherlands), and University Hospital Leuven (Leuven, Belgium). Selection of participating centers was based on availability of fecal samples in the study biobank. Samples from infants born in the period between December 2014 and December 2016 were selected for further analyses. Probiotics were not administered routinely in any of the participating centers. Infants with congenital gastrointestinal malformations (anus atresia, Hirshprung’s disease) and surgery of the gastro-intestinal tract were excluded. Additional exclusion criteria include the development of bacterial sepsis and/or meningitis (with both clinical signs of systemic infection and culture-derived bacterial pathogens from blood and/or cerebral spinal fluid (CSF)), diagnosis or suspicion for NEC (conform Bell’s criteria), and spontaneous intestinal perforation (SIP) [50]. Infants with insufficient fecal sample mass (<100 mg) on two or more of the predefined time points were excluded. The study was approved by the local institutional review boards of all participating centers (amendment A2016.363) and written informed consent was obtained from parents of included infants.

### 2.2. Study Groups

Included infants were categorized according to the variables of interest: GA and mode of delivery. Cases and controls were defined as born 24–26 6/7 and 27–29 6/7 weeks of gestation, respectively. From epidemiological data, it is known that the morbidity and mortality of infants is inversely correlated with the gestational age at birth, with larger week-to-week variations at earlier GA [51]. Assuming this would reflect on VOC patterns, but also taking into account the low incidence of birth at 24 and 25 completed weeks of gestation in the participating centers (ca. 4/year/center), the cut-off was arbitrarily set at 27 weeks. For mode of delivery, infants were assigned to the subgroup of (**1**) vaginally born infants or (**2**) infants born by C-section. Infants were matched exclusively based on the birth center.

### 2.3. Sample Size Calculation

We were not able to perform a sample size calculation due to lack of previous studies on this subject. Based on research on the effect of sampling conditions and enteral feeding type on VOC patterns, we assumed that a sample size of 15 subjects per group would suffice to identify clinically significant differences in VOC patterns [48,52].

### 2.4. Definitions

The enteral feeding practice was categorized as (**1**) predominantly consisting of mother’s milk (MM >75% of total daily enteral feeding volume consisted of raw or pasteurized own mother’s milk +/− pasteurized donor milk), (**2**) predominantly consisting of formula milk (FM > 75% of total daily enteral feeding volume consisted of formula milk) and (**3**) consisting of a combination of mother’s and formula milk (MM/FM). GA was defined as the number of weeks since the last maternal menstrual period. Age at full enteral feeding was defined as the first day of life at which infants were enterally fed > 120 mL/kg/day or did not receive parenteral feeding for over two consecutive days.

### 2.5. Sample Collection

Fecal samples were collected by the nurses at the participating NICUs from the diaper, placed in a container (Stuhlgefäß 10 mL, Frickenhausen, Germany), and subsequently stored at −20 °C within one hour after collection, until further handling. In case of multiple stool productions per day, the first fecal sample was stored. Sample collection was ceased in case of transfer to a referral hospital or decease before the postnatal age of 28 days. Fecal samples collected at 7 (t1), 14 (t2), and 21 (t3) (±2) days postnatally were selected for fecal VOC analysis.

### 2.6. VOC Analysis

**Figure 1:**
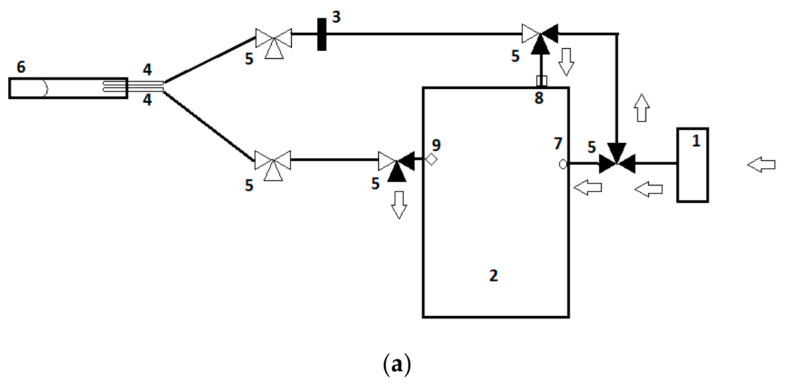

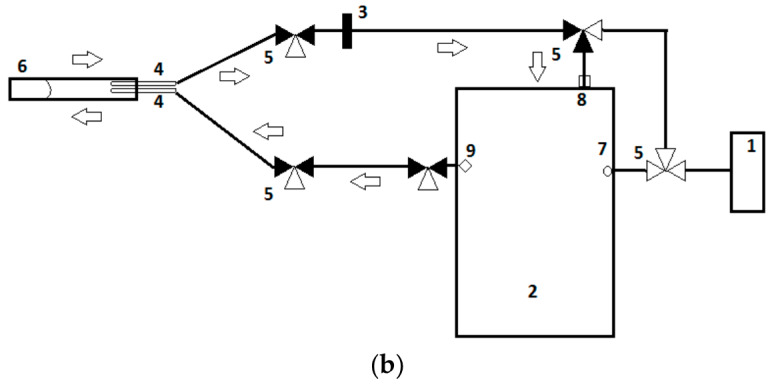
A schematic illustration of electronic nose setup during: (**a**) purging of the sensors and subsequently obtaining a baseline reference signal and (**b**) performing the actual sample measurement. A dark cone in a three-way valve (number 5) illustrates an air flow can pass, while a white cone prevents this. The arrows depict the air flow through the measurement setup. (**a**) First, the sensors are purged for 90” with filtered air derived from ambient air passing the A1-filter. The airflow, containing the residual volatile organic compounds (VOC), detached from the sensors, and is expelled through the exhaust port. Subsequently, a baseline reference signal is obtained in 30” using filtered air. (**b**) After the baseline reference signal is obtained, the actual measurement takes place in 60”. By rotating several three-way valves, a closed loop in connection with the fecal sample is formed (**6**). This loop prevents dilution of fecal VOCs with ambient air and, moreover, causes a continuous flow of fecal VOC passing the sensors. After the measurement, the three-way valves are rotated back to their original positions (Figure 1a) and the sensors are purged. (**1**) A1 filter, (**2**) Cyranose320^®^, (**3**) Polyethersulfone filter, (**4**) blunt needle, (**5**) three-way valve, (**6**) a vacutainer containing feces, (**7**) purge inlet, (**8**) sensor inlet, (**9**) exhaust portal, and (**10**) oxygen hose. Adapted with permission from Berkhout et al., 2016, Supplementary Material [54].

The VOC analysis method was analogous to previous studies conducted by our research group [48]. In short, fecal samples were analyzed for eight days within two consecutive weeks by means of an eNose device (Cyranose 320^®^, Smiths Detections, Pasadena, CA, USA). Approximately 150 mg sample mass was weighted on a calibrated scale (Mettler Toledo, AT 261 Delta Range, Columbus, OH, USA) and transferred into a sealed vacutainer (BD vacutainer, Belliver Industrial Estate, Plymouth, UK). Prior to analysis, samples were thawed to room temperature (18 °C) for 10 min, and subsequently connected to the eNose in an airtight loop system to prevent ambient air dilution (Figure 1a). The airtight system consisted of two needles (Terumo Europe N.V., Leuven, Belgium) pierced through the top of the vacutainer and connected to the eNose by a tube (Argyle Kendall tube, 3 mm, Mansfield, MA, USA). To control the airflow, a three-way stopcock system (BD Connecta, Helsinborg, Sweden) was used. The needles, tubes, and three-way stopcocks were replaced after each measurement to prevent contamination. To prevent condensation contamination of the eNose, a polyethersulfone syringe water filter (VWR International B.V., Arlington Heights, IL, USA) was added to the system. In between sample analysis, sensors were purged with filtered ambient air (VOC-filter, A1, North Safety, Middelburg, The Netherlands) in order to eliminate the remaining VOC on the sensors (Figure 1b). In addition, a baseline measurement was obtained by analyzing an empty vacutainer.

The applied eNose device allows for the differentiation of groups based on pattern recognition analysis. This pattern is recognized based on a nanocomposite array consisting of 32 polymer sensors. Each sensor has a unique polymer coating, which results in a competitive interaction between the sensors and VOC from the sample upon exposure. Subsequently, changes in electrical resistance in each sensor occur, depending on sensor material and chemical composition of the VOC. These changes are registered and result in a scent pattern that can be read out using the manufacturer’s software [53]. The specific VOC to which a particular sensor reacts belongs to the company’s proprietary and is not generally known.

### 2.7. Statistical Analysis

#### 2.7.1. Demographic and Clinical Data

Statistical analyses of demographic and clinical data were performed using Statistical Package for the Social Science (SPSS) version 26.0 (IBM Corp., Armonk, NY, USA). Where considered appropriate, Mann-Whitney U-test, Student’s *t*-test, Chi-Square test, or Fisher’s exact tests were used to compare study groups. Normally distributed continuous data are presented as mean and standard deviation, whereas non-normally distributed continuous data are presented as a median and interquartile range (IQR). Distribution of the data was visually assessed. Categorical data are presented as numbers and percentages. A *p*-value < 0.05 was considered statistically significant.

#### 2.7.2. eNose Data

The statistical analyses of VOC profiles in relation to GA and mode of delivery were conducted using R version 3.6.3 packages ‘stats’, ‘gplot’, and ‘global test’. The R script is made available in Supplement 1, Statistical analysis. First, the empirical distributions of the measurements per sensors were assessed. A variation in sensor output based on the measurement date was observed, a trend comparable across all sensors (Supplement 1, Statistical analysis, pages 6–10, boxplots s1–s32). We investigated if any of the clinical variables, namely gestational age, mode of delivery, birth weight, and enteral feeding type, could explain this trend, but there was no strong association explaining the day-to-day variation in the sensor’s output. Therefore, the sensor data were corrected for the date on which samples were measured. First, the date of measurement was read as a factor, and the 32 sensors’ outcome was read as numeric, continuous variables. The empirical data distribution of each sensor variable seemed to follow a continuous distribution, with each fitting a linear regression as response variables and with the measurement date presenting as an explanatory variable. The residuals of these regression models yield the new sensor data set with values corrected for the effect of measurement dates (cfr. Supplement 1, page 15). The original measurements are supplemented in Supplement 2.

In order to identify the association between fecal VOC profiles and the various study groups, the values representing the electrical resistance of each one of the 32 Cyranose^®^ polymer sensors, corrected by the previously mentioned linear regression model, were compared according to GA and mode of delivery. To do so, both an ANOVA (ANalysis of VAriances) was applied per sensor to compare the measurements between groups as well as a global test to compare measurements of all sensors between groups. For this analysis, GA was categorized using 27 weeks as a cut-off, creating two groups, so that both it and the mode of delivery are binary variables. As such, the global test with a logistic link function was used. Including all 32 sensors’ data, this test allows for the assessment of changes in sensor data patterns between groups of GA on the one hand, and mode of delivery on the other hand. Results of the F-test from a one-way ANOVA were reported for the sensor with the lowest *p*-value on a given time point.

## 3. Results

### 3.1. General Characteristics

**Figure 2:**
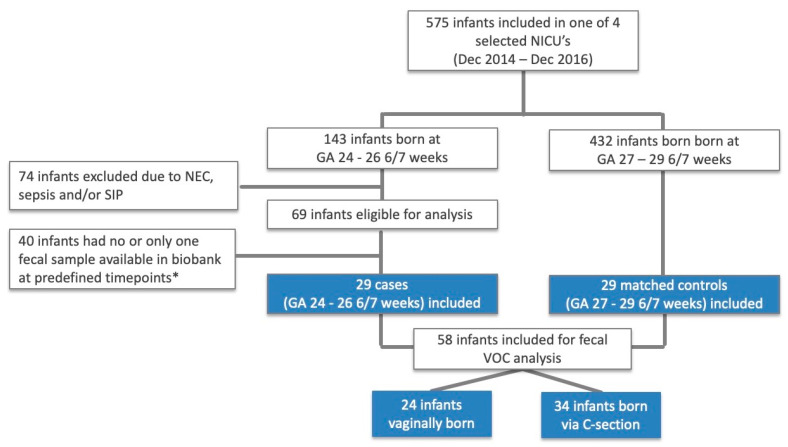
Flow of participants. Abbreviations: GA, gestational age. * Day of life 7 (+/− 2 days), 14 (+/− 2 days), and 21 (+/− 2 days).

Fifty-eight infants were included in the study of which 29 were born at GA 24–26 6/7 weeks’ gestation and 24 were born vaginally (Figure 2). Birth weight was significantly lower in infants born at GA 24–26 6/7 weeks, compared to infants born from 27 to 29 6/7 weeks of gestation. None of the included neonates had developed sepsis (early-/late-onset), NEC, SIP, or bacterial meningitis. One neonate (GA <27 weeks, vaginally delivered) was suspected of meningitis and was treated with broad spectrum antibiotics (meropenem) for 20 days, but was never formally diagnosed with an invasive bacterial infection, given the repeatedly negative blood and CSF cultures. All but four children received antibiotics in the first three weeks of life, of whom 39 were exposed to antibiotics for longer than two days, and 28 received one or more courses after the first week of life. The overall median duration of antibiotic exposure was five days (IQR 3–7). Infants born at GA < 27 weeks were exposed for a longer time, compared to infants born after 27 weeks of gestation (Table 1). Further demographic and clinical data are depicted in Table 1 and Table 2, according to GA and delivery mode, respectively, with no other statistically significant differences (raw data available in Supplement 2).

### 3.2. Influence of Gestational Age and Delivery Mode on Fecal VOC

Fecal VOC profiles, as measured by the Cyranose^®^ eNose, did not differ significantly in the first three weeks of life between infants born at 24–26 6/7 weeks and those born at 27–29 6/7 weeks of gestation (Supplement 1, Figures S1, S3 and S5, Heatmaps for corrected sensor data by gestational age at day 7, 14, and 21, resp.). Similarly, VOC profiles did not differ between infants born vaginally and via C-section (Supplement 1, Figures S2, S4, and S6, Heatmaps for corrected sensor data by mode of delivery at day 7, 14, and 21, resp.). These results were consistent when combining all sensors together (global test) and analyzing each sensor separately (F-test) (Table 3).

## 4. Discussion

In the current study, the potential influence of GA and delivery mode on VOC outcome was assessed. In our cohort of preterm infants, longitudinal fecal VOC profiles up to three weeks of postnatal age were not significantly influenced by GA or mode of delivery, when measured by an eNose device.

### 4.1. Influence of Gestational Age on Fecal VOC

To our knowledge, previous studies investigating the effect of GA on metabolomics in preterm neonates are only performed within the first week of life [55]. Available microbiota research in the first months postnatally shows that fecal composition is influenced by GA, but mainly after ca. 30 weeks postmenstrual age (PMA) [44,56]. Between 24 and ca. 30 weeks’ PMA, regardless of the GA at birth, the microbiota predominantly consists of *Bacili*, while *Gammaproteobacteria* become more abundant after 29 to 30 weeks [56]. This PMA term is also associated with an increased development of immune-competent intestinal Paneth cell’s, which change the gut metabolism and, hypothetically, fecal VOC patterns [57].

Our cohort consisted of two groups, born at 24–26 and 27–29 completed weeks of gestation and fecal samples were compared on predefined time points, based on postnatal day of life, rather than PMA. It is, therefore, possible that stratification of patients according to PMA at sample collection, rather than GA at birth, would influence fecal VOC patterns, similar to the findings in microbiota studies [44,56]. This hypothesis should be investigated in future research.

An additional explanation for our results is that potentially only a weak effect of GA exists on fecal VOC, which is undermined by other factors to which the very preterm infants in our cohort are exposed, such as broad spectrum antibiotics and increased oxidative stress, caused by, e.g., BPD and intraventricular hemorrhage (IVH) [38,58].

### 4.2. Influence of Mode of Delivery on Fecal VOC

The second aim of this study was to compare the longitudinal course of fecal VOC patterns between vaginal birth and birth via C-section in the same cohort. Our results suggest that mode of delivery does not affect VOC profiles significantly during the first weeks of life in infants born at GA <30 weeks. To our knowledge, no previous metabolic research has been conducted on this topic, but several microbiome studies are available [59,60]. In line with our findings on fecal VOC, mode of delivery was reported not to impact gut microbiota diversity and composition in the first 100 days of life of preterm infants (GA <32 weeks), while, in infants born prior to 37 weeks of gestation, the delivery mode influenced the microbiota composition in the first week, but not in the second and third week postnatally [59,60].

This is in contrast with studies on term infants, which show short- and long-term differences in the microbiome community structure and function after birth via C-section when compared to vaginally born infants [45,61]. It is hypothesized that antibiotic exposure, oxidative stress, and environmental factors inherent to NICU hospitalization would have a more dominant effect on the gut microbial community, and potentially on VOC signals, than mode of delivery [62].

### 4.3. Strengths and Limitations

The first strength of this study is the prospective and standardized collection and handling of fecal samples by which we reduce the risk of potential pre-analytical errors [48,52,54]. A second advantage is that we have longitudinal samples from cases that were strictly matched to controls based on center of birth, and, thus, indirectly by center-specific treatment protocols. By this approach, we avoid a non-random distribution of factors already known for their influence on VOC, such as feeding type and center-specific environmental factors [48,54]. This is also expressed in the homogeneity in distribution of these variables between groups (Table 1 and Table 2). Lastly, by excluding infants with congenital gastrointestinal malformations and infants who developed LOS, NEC, SIP, and early onset sepsis, we exclude the measurement of disease-specific VOC.

There are several limitations that need to be addressed. First, within the group of mother’s milk-fed children, it was decided not to make a distinction between infants receiving raw own mother’s milk (OMM) and those receiving pasteurized own mother’s or pasteurized donor human milk (resp. pOMM and DHM). Based on previous studies, a (mildly) different microbiota, and potentially VOC profile, could be supposed [46]. Yet, we do not expect this to have significantly influenced the VOC patterns in this study as, based on in-house protocols, the proportion of infants receiving predominantly DHM or pOMM is estimated to be very small. In three of four participating centers, DHM was only administered in case of insufficient OMM production, while, in the fourth center, OMM was only pasteurized if pathogens were cultured in an OMM sample.

An additional limitation is that our exclusion criteria did not include non-infectious diseases with a potential effect on the infant’s metabolic state, such as BPD, severe IVH, and patent ductus arteriosus (PDA) [38,58,63,64]. The role of IVH and PDA in a VOC outcome has not yet been established and should be further investigated.

## 5. Conclusions

Our results show that VOC profiles, as measured by an eNose device, in preterm infants born at GA <30 weeks, are not influenced by GA or mode of delivery during the first three weeks of life. If reproduced in other cohorts, these results implicate that it would be methodologically appropriate not to correct for GA and mode of delivery when performing fecal VOC research in a preterm population born before 30 weeks’ gestation until three weeks postnatally. We hypothesized that environmental factors (e.g., enteral feeding type and medication exposure) and clinical conditions (e.g., BPD and IVH) are likely to influence fecal VOC outcome to a greater extent than GA and delivery mode in this particular population. When not yet investigated, these variables should be addressed in further research.

## Figures and Tables

**Table 1:** Baseline characteristics for included neonates, based on gestational age (GA).

	24–26 Weeks (n = 29)	27–29 Weeks (n = 29)	*p*-Value
Included neonates t1, t2, t3 (n)	23, 27, 27	24, 27, 26	0.98
GA days (mean [SD])	184 [5]	199 [5]	<0.001 *
Gender female (n[%])	16 [55]	15 [48]	0.60
Birth weight grams (mean [SD])	856 [140]	1166 [198]	<0.001 *
Mode of delivery vaginal (n[%])	12 [41]	12 [41]	1.00
Center of birth (n [%])			1.00
1	7 [24]	7 [24]	
2	6 [21]	6 [21]	
3	8 [28]	8 [28]	
4	8 [28]	8 [28]	
Feeding mode prior to t1 (n[%])			0.28
Mother’s milk	12 [52]	7 [29]	
Formula milk	4 [17]	6 [25]	
Combination MM/FM	7 [30]	11 [46]	
Feeding mode prior to t2 (n[%])			0.50
Mother’s milk	21 [78]	24 [89]	
Formula milk	3 [11]	2 [7]	
Combination MM/FM	3 [11]	1 [4]	
Age at full enteral feeding days (mean [SD])	14 [3]	14 [2]	0.25
Feeding mode prior to t3 (n[%])			0.58
Mother’s milk	20 [74]	21 [84]	
Formula milk	3 [11]	1 [4]	
Combination MM/FM	4 [15]	3 [12]	
Parental feeding days (median [IQR])			
Prior to t1	7 [6–7]	7 [6–7]	0.53
Prior to t2	11 [9–14]	12 [9–13]	0.99
Prior to t3	11 [9–14]	12 [11–13]	0.27
Antibiotic exposure prior to t3 (n[%])	27 [100]	22 [85]	0.05
Antibiotic exposure days (median [IQR])			
Prior to t1	3 [2–4]	3 [2–4]	0.22
Prior to t2	4 [2–6]	3 [2–5]	0.04 *
Prior to t3	5 [3–8]	3 [2–6]	0.03 *
Invasive ventilation prior to t3 (n[%])	13 [48]	6 [23]	0.09
Invasive ventilation days (median [IQR])			
Prior to t1	5 [1–6]	3 [2–4]	0.33
Prior to t2	6 [4–12]	3 [2–5]	0.10
Prior to t3	7 [4–16]	3 [2–6]	0.11
Sample weight grams (median [IQR])			
t1	149 [128–163]	151 [137–163]	0.22
t2	154 [146–161]	152 [137–158]	0.68
t3	155 [141–162]	148 [137–157]	0.34
Sample age months (median [IQR])			
t1	35 [32–50]	35 [33–44]	0.82
t2	35 [31–46]	35 [33–44]	0.73
t3	35 [32–45]	35 [33–45]	0.78

**Abbreviations:** n, number. SD, standard deviation. IQR, interquartile range. t1, day of life 7. t2, day of life 14. t3, day of life 21. MM, mother’s milk. FM formula milk. * *p*-value < 0.05.

**Table 2:** Baseline characteristics for infants compared based on birth weight.

	Vaginal (n = 24)	C-Section (n = 34)	*p*-Value
Included neonates t1, t2, t3 (n)	21, 24, 21	26, 30, 32	0.84
GA in days (mean [SD])	190 [10]	192 [9]	0.29
Gender female (n [%])	12 [50]	18 [53]	0.83
Birth weight grams (mean [SD])	1055 [237]	980 [225]	0.23
Center of birth (n [%])			0.05
1	8 [33]	6 [18]	
2	6 [25]	6 [18]	
3	2 [8]	14 [41]	
4	8 [33]	8 [24]	
Feeding mode prior to t1 (n[%])			0.93
Mother’s milk	9 [43]	10 [39]	
Formula milk	4 [19]	6 [23]	
Combination MM/FM	8 [38]	10 [39]	
Feeding mode prior to t2 (n[%])			0.23
Mother’s milk	18 [75]	27 [90]	
Formula milk	4 [17]	1 [3]	
Combination MM/FM	2 [8]	2 [7]	
Feeding mode prior to t3 (n[%])			0.27
Mother’s milk	14 [70]	27 [84]	
Formula milk	3 [15]	1 [3]	
Combination MM/FM	3 [15]	4 [13]	
Age at full enteral feeding days (median [IQR])	14 [12–17]	14 [12–16]	0.83
Parental feeding days (median [IQR])			
Prior to t1	7 [7–7]	7 [6–7]	0.25
Prior to t2	11 [10–13]	11 [9–14]	0.84
Prior to t3	11 [11–13]	11 [9–14]	0.62
Antibiotic exposure prior to t3 (n[%])	20 [95]	29 [91]	1.00
Antibiotic exposure days (median [IQR])			
Prior to t1	3 [2–3]	3 [2–4]	0.67
Prior to t2	3 [2–6]	4 [2–5]	0.70
Prior to t3	4 [2–7]	5 [2–6]	0.60
Invasive ventilation prior to t3 (n [%])	7 [33]	12 [38]	0.76
Invasive ventilation days (median [IQR])			
Prior to t1	5 [2–5]	4 [1–6]	0.95
Prior to t2	6 [4–12]	4 [1–7]	0.28
Prior to t3	11 [4–19]	5 [2–7]	0.34
Sample weight grams (median [IQR])			
t1	149 [128–159]	151 [132–163]	0.42
t2	154 [139–162]	152 [141–158]	0.30
t3	154 [131–162]	149 [141–162]	0.98
Sample age months (median [IQR])			
t1	36 [32–46]	34 [33–45]	0.91
t2	35 [31–44]	35 [33–45]	0.94
t3	35 [32–44]	35 [32–44]	0.73

**Abbreviations:** n, number. SD, standard deviation. IQR, interquartile range. t1, day of life 7. t2, day of life 14. t3, day of life 21.

**Table 3:** F test for one-way ANOVA and global test for gestational age and mode of delivery at each predefined time point.

	*p*-Value (t1)	*p*-Value (t2)	*p*-Value (t3)
Gestational age			
F test for ANOVA *	0.36	0.13	0.61
Global test	0.38	0.65	0.96
Mode of delivery			
F test for ANOVA *	0.52	0.50	0.27
Global test	0.72	0.95	0.33

**Abbreviations:** t1, day of life 7. t2, day of life 14. t3, day of life 21. * For the F-test, only the smallest *p*-values across all sensors is reported.

## Supplementary Materials

The following are available online at https://www.mdpi.com/2079-6374/10/5/50/s1, Supplement 1: Statistical analysis: R script, including Figures S1, S3, and S5: Heatmap for corrected sensor data by gestational age at day 7, 14, and 21 of life, respectively. Figures S2, S4, and S6. Heatmap for corrected sensor data by mode of delivery at resp. day 7, 14, and 21 of life, respectively. Available online at https://www.mdpi.com/2079-6374/10/5/50/s2, Supplement 2: Raw data of the current study with clinical characteristics and sensor data output.

Supplementary File 1

Supplementary File 2
