# Supplementary material for: Fecal Volatile Organic Compound Profiles are Not Influenced by Gestational Age and Mode of Delivery: A Longitudinal Multicenter Cohort Study
Source: Biosensors (Basel). 2020 May 11;10(5):50. doi: 10.3390/bios10050050 (PMC7277672; doi:10.3390/bios10050050)
Supplement: Supplementary File 1 [file biosensors-10-00050-s001.pdf]

Supplement 1: Statistical analysis

Fecal Volatile Organic Compound Profiles Are Not Influenced By Gestational Age and Mode of Delivery: A Longitudinal Multicenter Cohort Study

Renee Menezes

2020-03-24

```
myname <- "enose"
name.folder <- "elmanouni sofia"

mynam <- "/media/renee/Seagate Expansion Drive/"
mydir.base <- paste(mynam, "Projects/", sep="")
mydir <- paste(mydir.base, name.folder, sep="")

mydir.scripts <- paste(mydir, "scripts", sep="/")
mydir.data <-  paste(mydir, "data", sep="/")
mydir.output <-  paste(mydir, "output", sep="/")

setwd(mydir.scripts)

source(paste(mydir.base,"functions_wilcoxon_t_logistic
_fdr.R",sep="/")) # loads functions for FDR and graphs
source(paste(mydir.base,"mysplit.R",sep="/")) # loads
functions for mt cor and graphs
source(paste(mydir.base,"plot_density_percolumn.R",sep
="/")) # function to make graphs of densities per colu
mn of a data matrix, with proper limits
source(paste(mydir.base,"var_in_colour.R",sep="/")) #
loads functions for mt cor and graphs

library(gplots)
#library(edgeR)
library(globaltest)
```

```
data.counts <- read.delim(paste(mydir.data, "/
              Supplement 1 .csv",
                                sep=""), sep=";")
#pdata <- read.delim(paste(mydir.data, "/pdata.txt", s
ep=""))
data.ann <- data.counts[, 1:11]
data.sens  <- as.matrix(data.counts[, 12:ncol(data.cou
nts)])
```

The data read contains 154 cases measured with 32 sensors, plus 11 variables.

We start by looking at the empirical distributions of the measurements per sensor:


```
pdensity.column(data.sens)
```

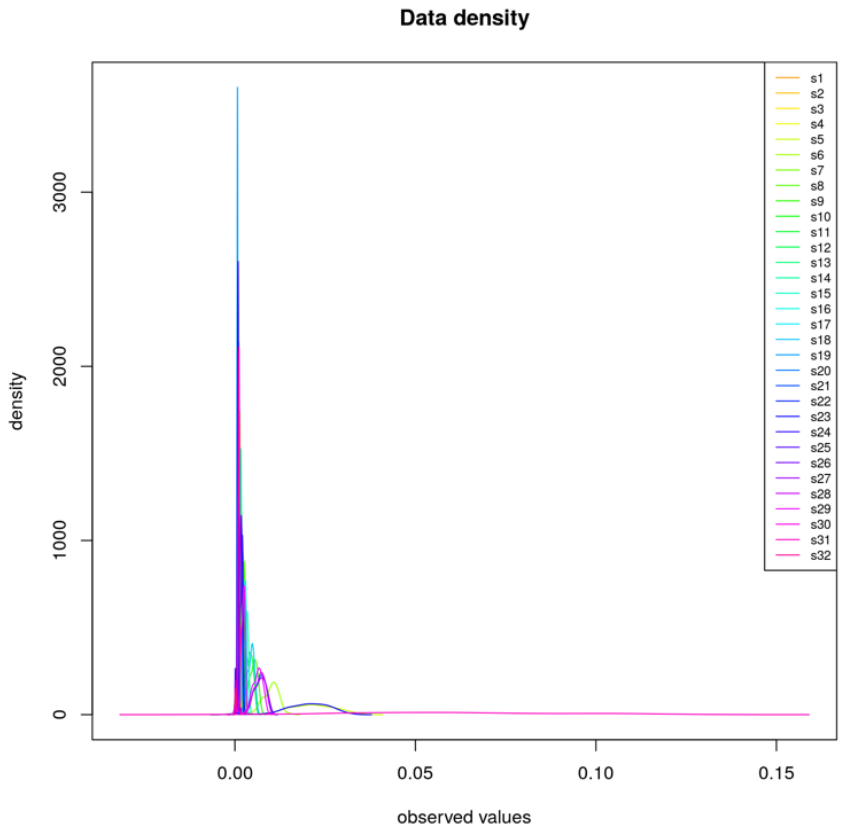

One sensor has much larger values than the others, as we can confirm in the plot of the maxima below:

```
plot(apply(data.sens, 2, max), pch=20, col=rainbow(nco
l(data.sens), start=0.1, end=0.9),
     xlab = "Sensor")
```

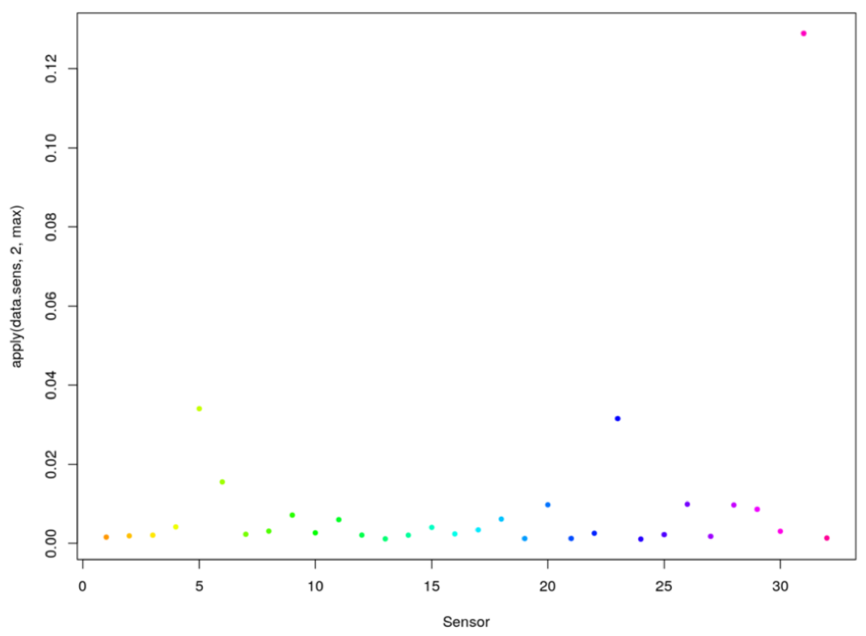

All measurements of this sensor have large values, as is illustrated by the histogram below:

```
hist(data.sens[, "s31"], col = "blue", main = "S31", x
lab = "measurements")
```

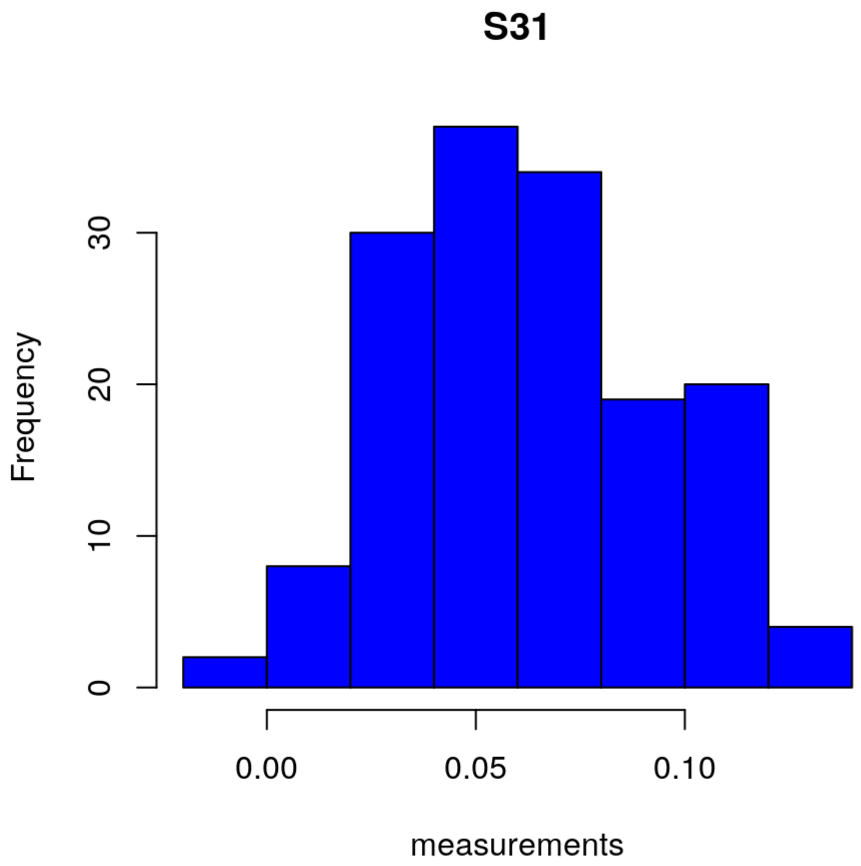

We now replot the empirical densities by fixing the x-axis maximum as the maximum of all data without measurements of the sensor with extreme values (31).

```
max.x <- max(data.sens[, -31])
pdensity.column(data.sens, xlim = c(0, max.x))
```

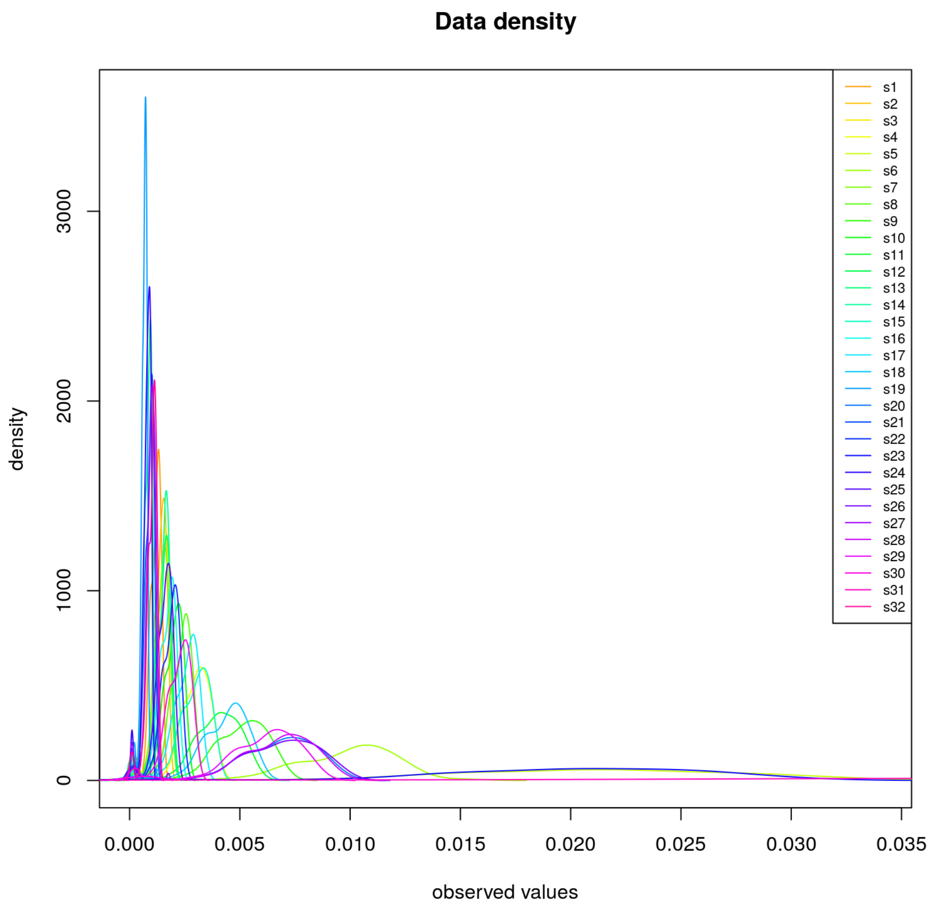

The best maximum seems to be around 0.01, as values above it are rare and do not discriminate between sensors.

Using the value of 0.01 as maximum, we check whether or not there is evidence of an association between the sensor data and clinical variables, in particular the date the sample was collected. The number of different dates, and number of cases collected on these dates, are:

```
table(data.ann$Date_of_sample_measurement)
```

```
##
## 15-1-2019 16-1-2019 17-1-2019 21-1-2019 22-1-2019 2
4-1-2019 25-1-2019 28-1-2019
##        45        24        28         8        20
18         2         8
```

```
myvar <- var.in.colour(data.ann$Date_of_sample_measure
ment)
heatmap.2(data.sens, trace = 'none', col = 'bluered',
breaks = seq(0, 0.01, by =0.001),
          RowSideColors = myvar[[1]])
legend("topright", legend = myvar[[3]], fill = myvar[[
2]], cex=0.5)
```

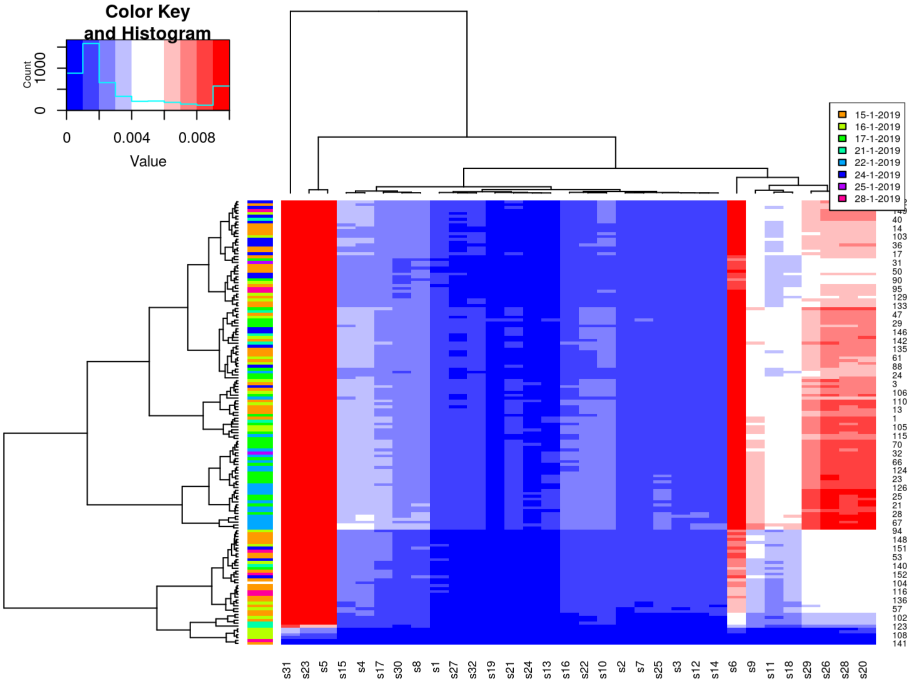


```
# Note that large values of sensor 31 can be explained
by the collection date. Indeed:

par(las=2)
cols <- rainbow(nlevels(data.ann$Date_of_sample_measur
ement), start = 0.5, end = 0.7)
plot(data.ann$Date_of_sample_measurement, data.sens[,
"s31"], col = cols)
```

Now examining the relationship between measurements per sensor and date:

```
par(las=2, mfrow = c(1, 2))
cols <- rainbow(nlevels(data.ann$Date_of_sample_measur
ement), start = 0.5, end = 0.7)
for(xc in 1:ncol(data.sens))
  plot(data.ann$Date_of_sample_measurement, data.sens[
, xc],
        col = cols, main = paste(colnames(data.sens)[xc
]))
```

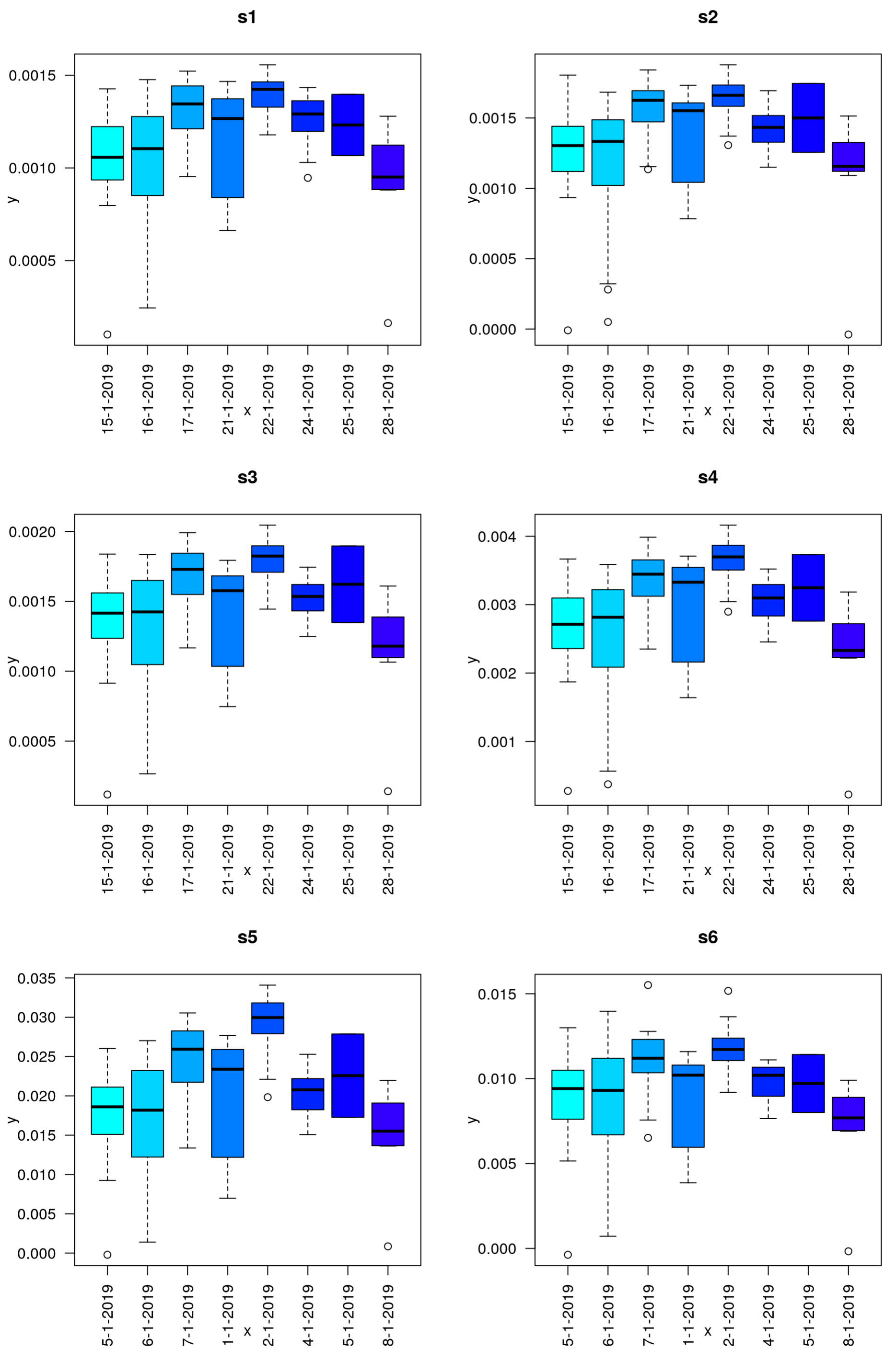

s7

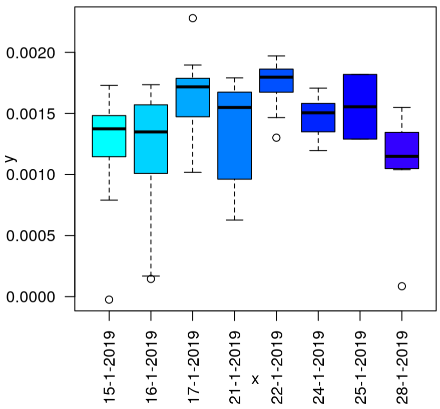

s8

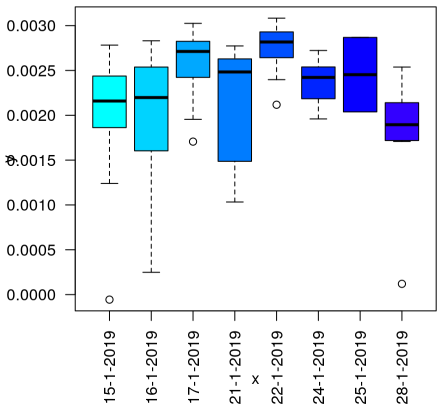

s9

s10

s11

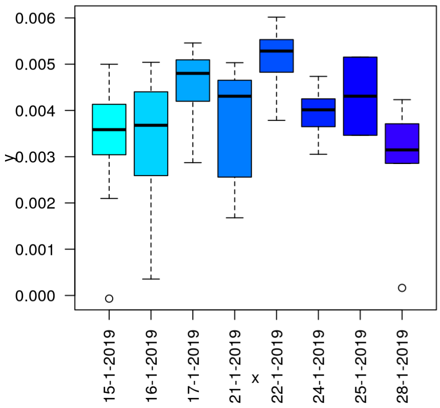

s12

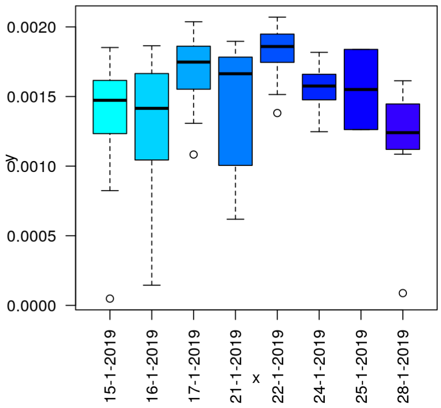

s13

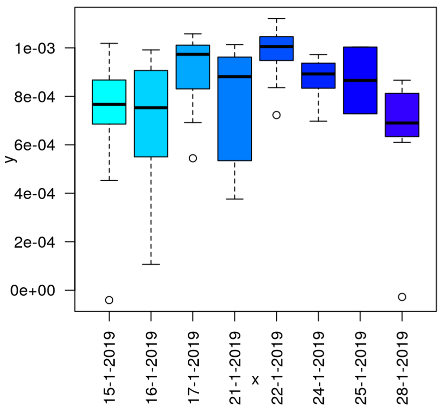

s14

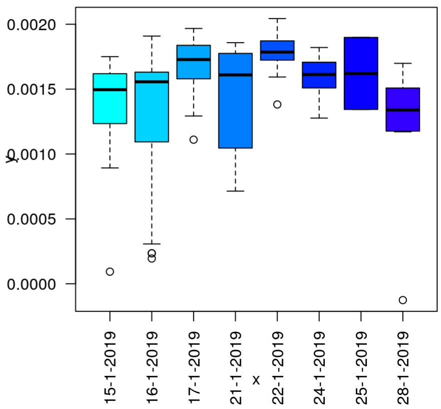

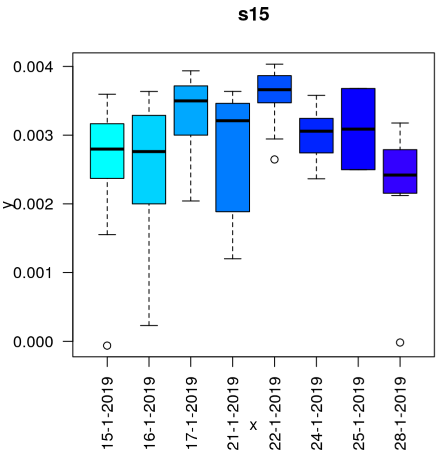
s15
y
x
15-1-2019
16-1-2019
17-1-2019
21-1-2019
22-1-2019
24-1-2019
25-1-2019
28-1-2019

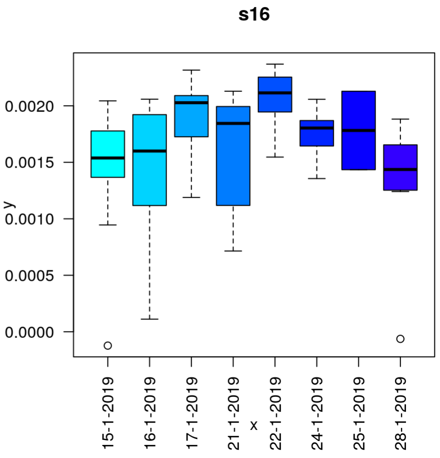
s16
y
x
15-1-2019
16-1-2019
17-1-2019
21-1-2019
22-1-2019
24-1-2019
25-1-2019
28-1-2019

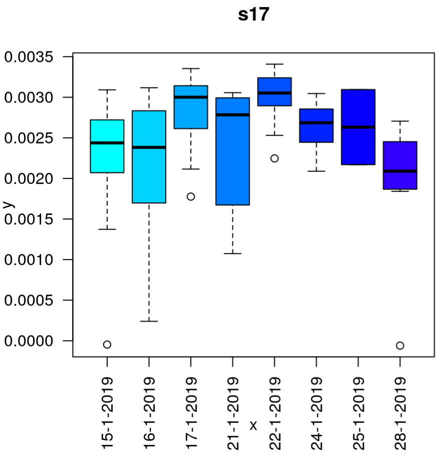
s17
y
x
15-1-2019
16-1-2019
17-1-2019
21-1-2019
22-1-2019
24-1-2019
25-1-2019
28-1-2019

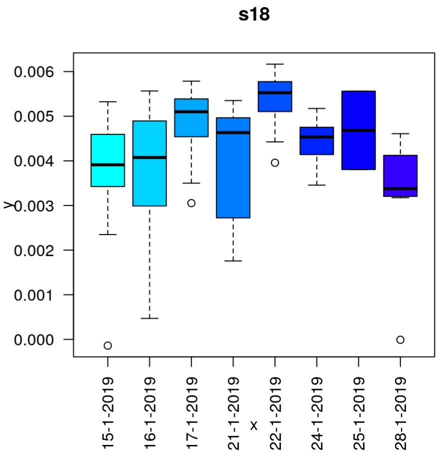
s18
y
x
15-1-2019
16-1-2019
17-1-2019
21-1-2019
22-1-2019
24-1-2019
25-1-2019
28-1-2019

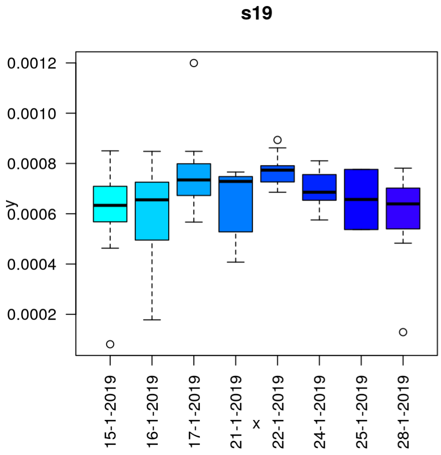
s19
y
x
15-1-2019
16-1-2019
17-1-2019
21-1-2019
22-1-2019
24-1-2019
25-1-2019
28-1-2019

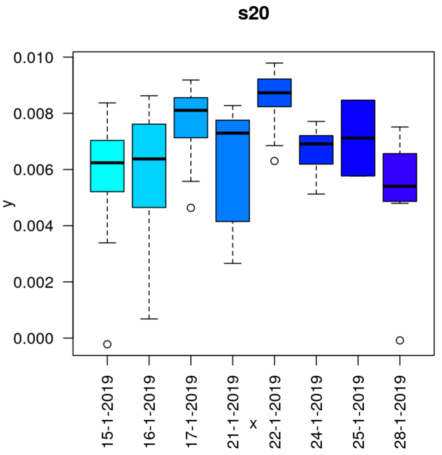
s20
y
x
15-1-2019
16-1-2019
17-1-2019
21-1-2019
22-1-2019
24-1-2019
25-1-2019
28-1-2019

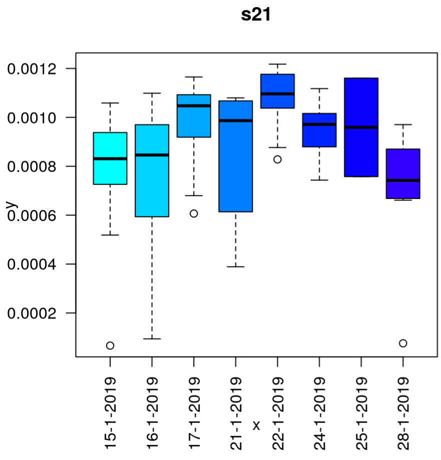
s21
y
x
15-1-2019
16-1-2019
17-1-2019
21-1-2019
22-1-2019
24-1-2019
25-1-2019
28-1-2019

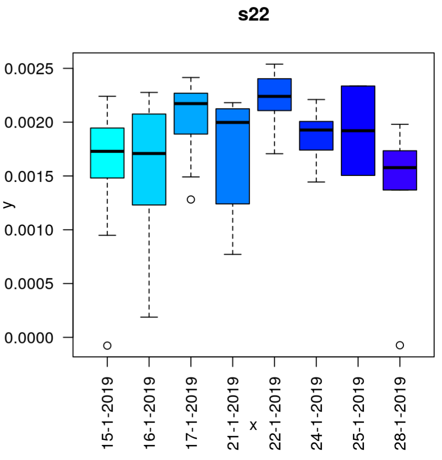
s22
y
x
15-1-2019
16-1-2019
17-1-2019
21-1-2019
22-1-2019
24-1-2019
25-1-2019
28-1-2019

s23

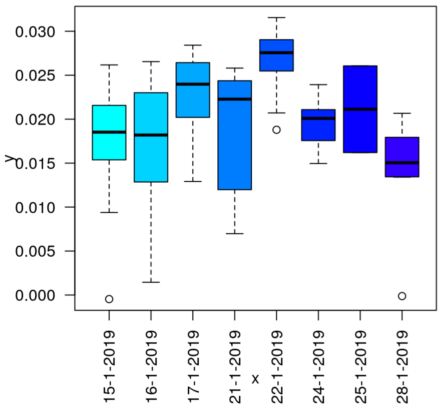

s24

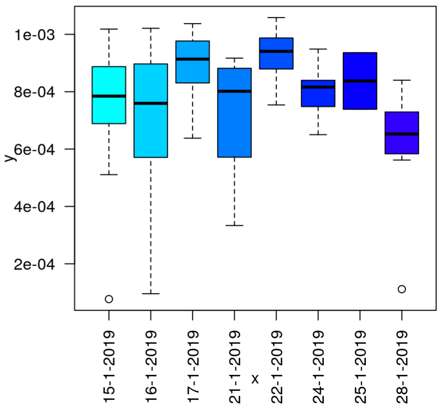

s25

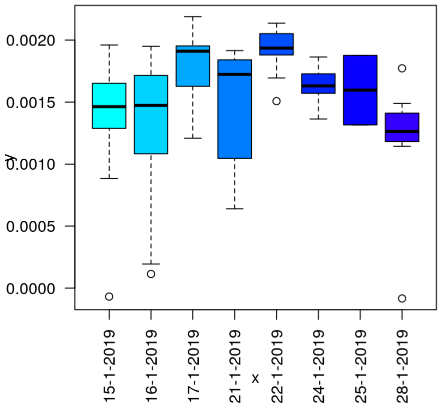

s26

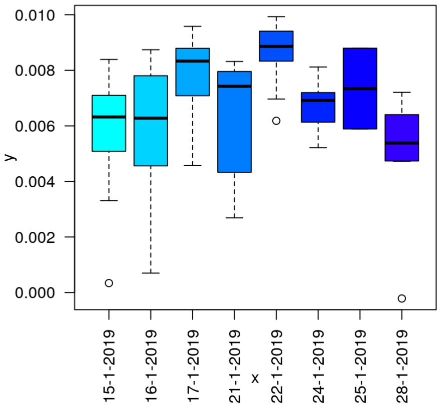

s27

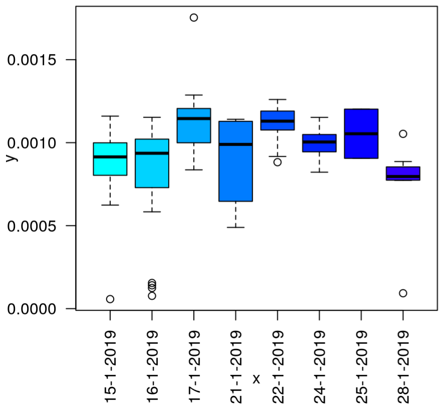

s28

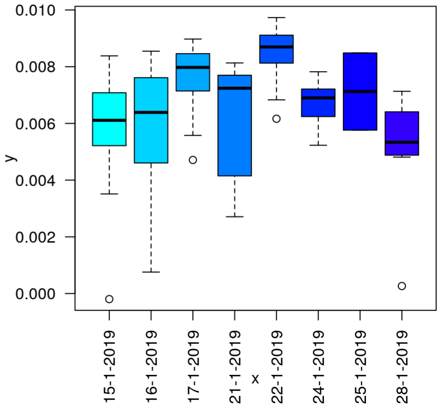

s29

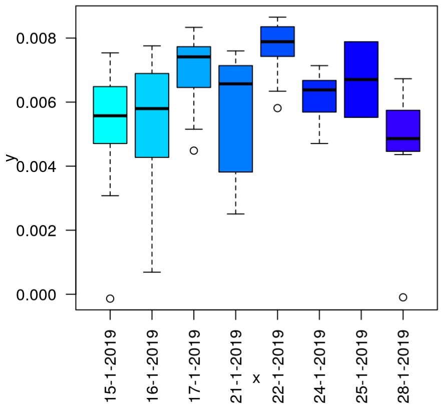

s30

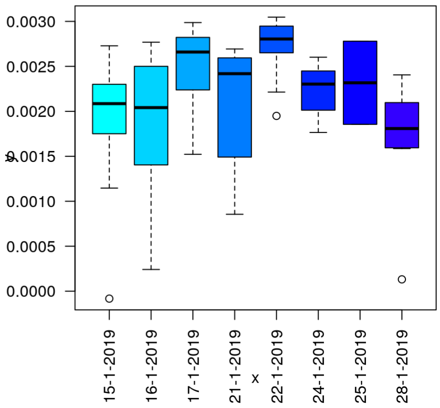

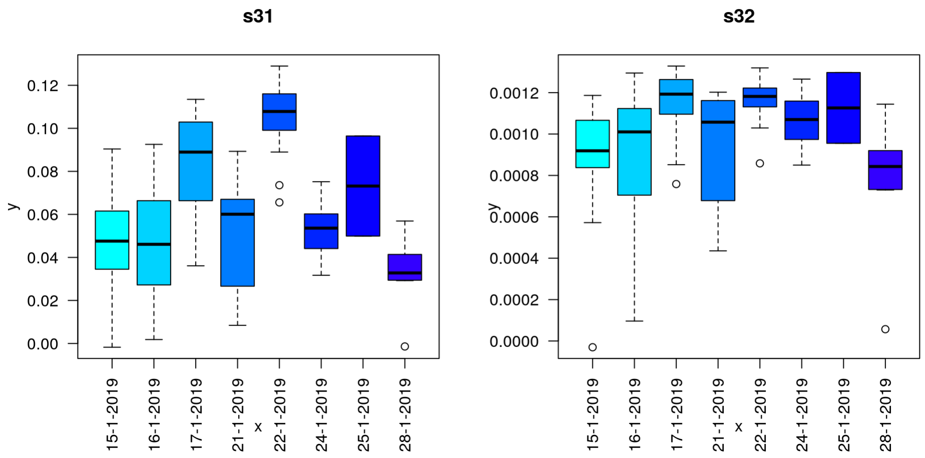

From the above, we see that the date indeed explains variability from sensor measurements. Specifically, we see that the mean measurement is sensor-specific, but that the trend is similar across sensors, with all sensors displaying the same trend between dates.

Other variables that may explain the split in samples are considered below:

```
f.gesage <- factor(cut(data.ann$Gestational_age_days,
breaks = c(0, 27*7, 30*7 )),
                   labels = c("<= 27w",  "> 27w"))
f.deliv <- factor(data.ann$Mode_of_delivery, labels =
c("vaginal", "c-section"))
f.bw <- factor(cut(data.ann$Birth.weight_grams, breaks
= c(0, 900, 1200, 2000)),
               labels = c("<= 900g", "900g < x <= 1200
g","> 1200g"))
data.ann$Feeding_mode_prior_day_of_life_analyzed[ data
.ann$Feeding_mode_prior_day_of_life_analyzed < 0] <- N
A
f.feed <- factor(data.ann$Feeding_mode_prior_day_of_li
fe_analyzed,
                 labels = c(">= 75% breast milk", ">=
75% formula milk", "combination"))
```

```
myvar <- var.in.colour(f.gesage, mystart=0.4, myend=0.
7)
heatmap.2(data.sens, trace = 'none', col = 'bluered',
breaks = seq(0, 0.01, by =0.001),
          RowSideColors = myvar[[1]])
legend("topright", legend = myvar[[3]], fill = myvar[[
2]], cex=0.5)
```

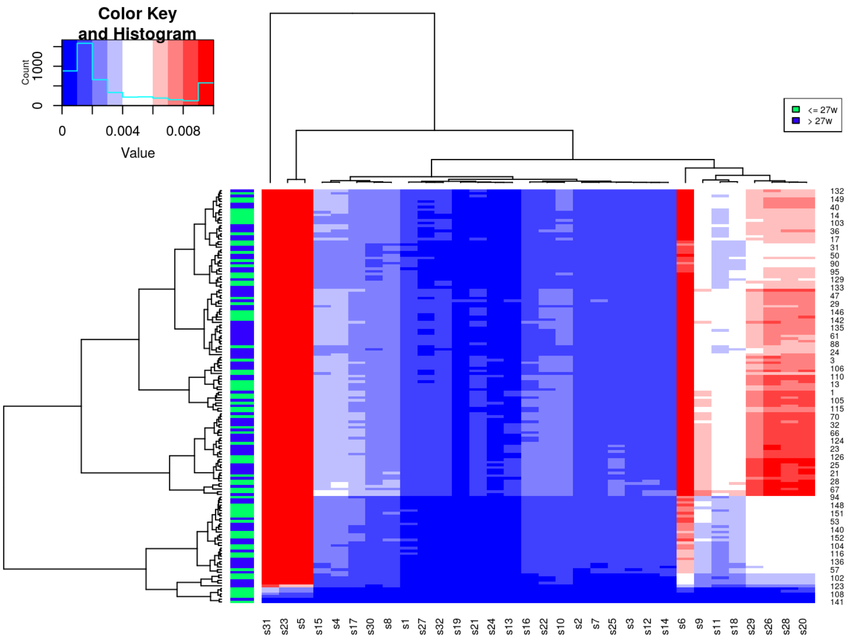


```
myvar <- var.in.colour(f.deliv, mystart=0.4, myend=0.7
)
heatmap.2(data.sens, trace = 'none', col = 'bluered',
breaks = seq(0, 0.01, by =0.001),
            RowSideColors = myvar[[1]])
legend("topright", legend = myvar[[3]], fill = myvar[[
2]], cex=0.5)
```

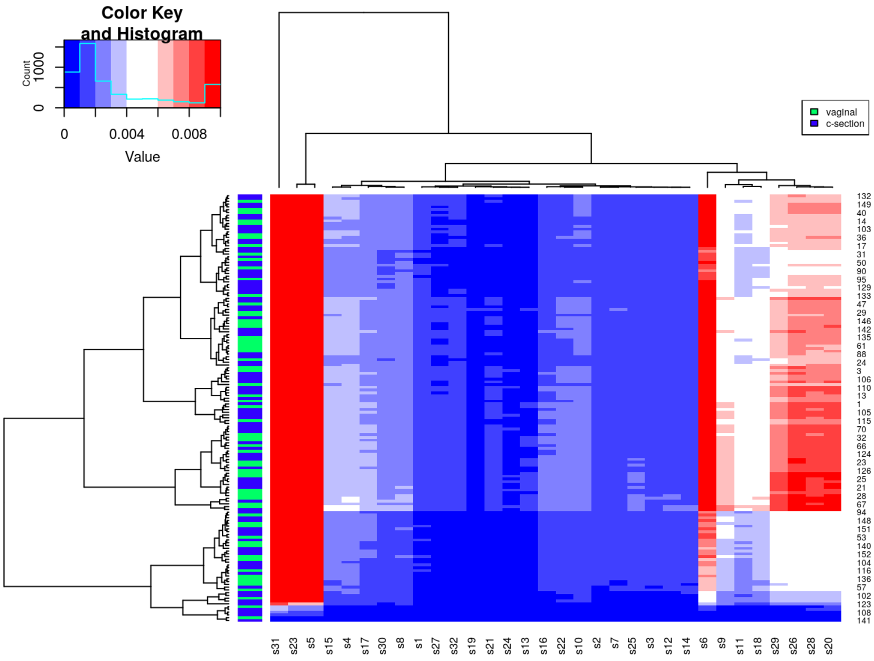


```
myvar <- var.in.colour(f.bw, mystart=0.4, myend=0.7)
heatmap.2(data.sens, trace = 'none', col = 'bluered',
breaks = seq(0, 0.01, by =0.001),
            RowSideColors = myvar[[1]])
legend("topright", legend = myvar[[3]], fill = myvar[[
2]], cex=0.5)
```

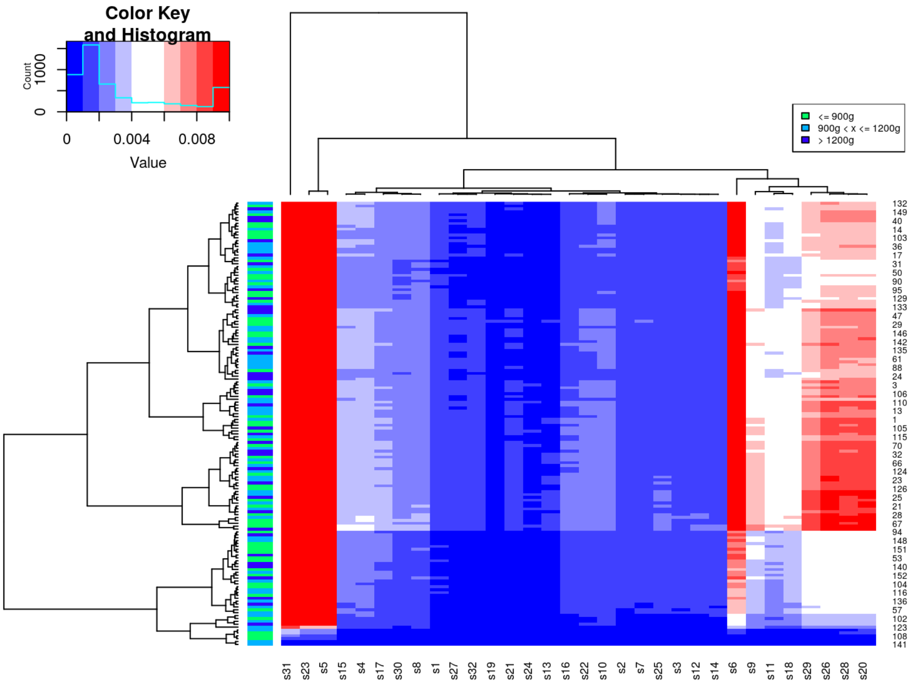


```
myvar <- var.in.colour(f.feed, mystart=0.4, myend=0.7)
heatmap.2(data.sens, trace = 'none', col = 'bluered',
breaks = seq(0, 0.01, by =0.001),
          RowSideColors = myvar[[1]])
legend("topright", legend = myvar[[3]], fill = myvar[[
2]], cex=0.5)
```

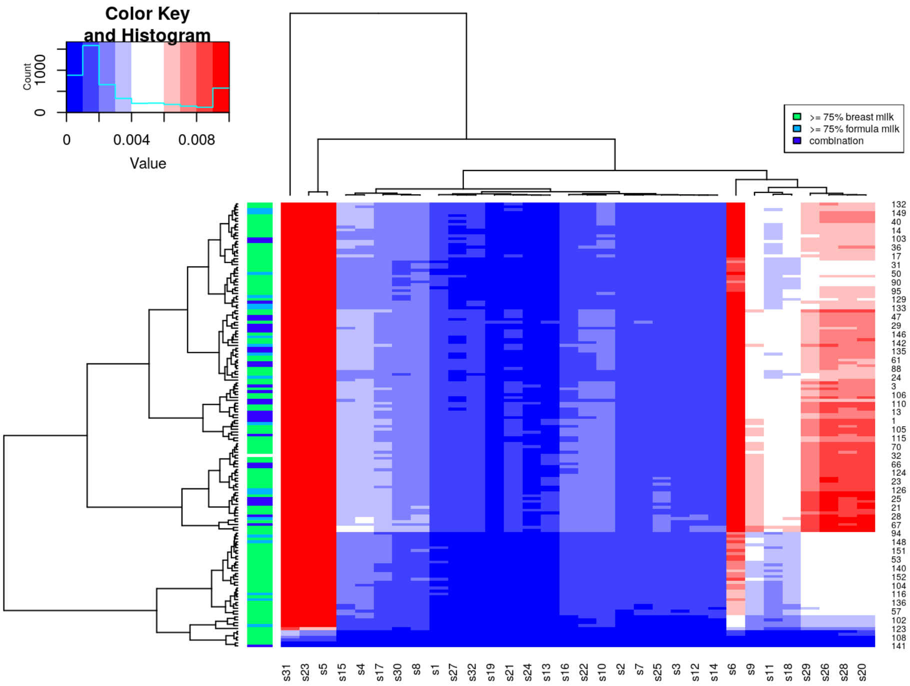

Gestational age is possibly the best variable to define a control group, namely using the cases with gestational age closest to “normal”. We check the observed gestational ages via a histogram:

```
normal.age <- 37*7 # normal gestational age (days)
hist(data.ann$Gestational_age_days, col = "blue", brea
ks = 15)
```

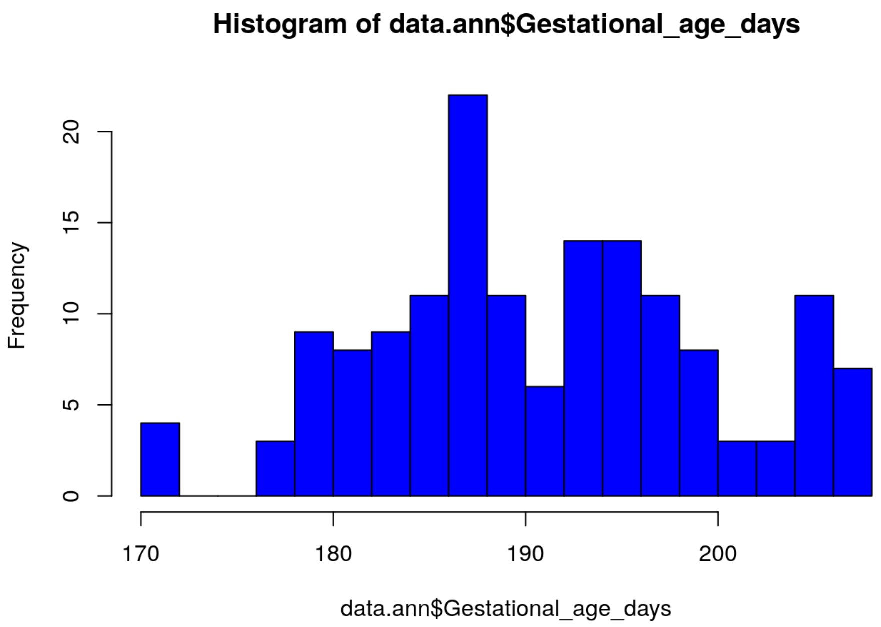

Note that the longest observed gestational age, 208, is considerably smaller than a gestational age of 37 weeks, or 259.

We now remake boxplots of sensor measurements according to measurement date using only cases with gestational age >27 weeks. Here we want to verify that the trend seen in the data is well captured by the cases with longer gestational age, so that these can be used as reference.

```
sel27 <- f.gesage == "> 27w"
par(las=2, mfrow = c(1, 2))
for(xc in 1:ncol(data.sens))
  plot(data.ann$Date_of_sample_measurement[sel27], dat
a.sens[sel27, xc],
       col = cols, main = paste(colnames(data.sens)[xc
]))
```

Only boxplots of sensor 31 and sensor 32 shown.

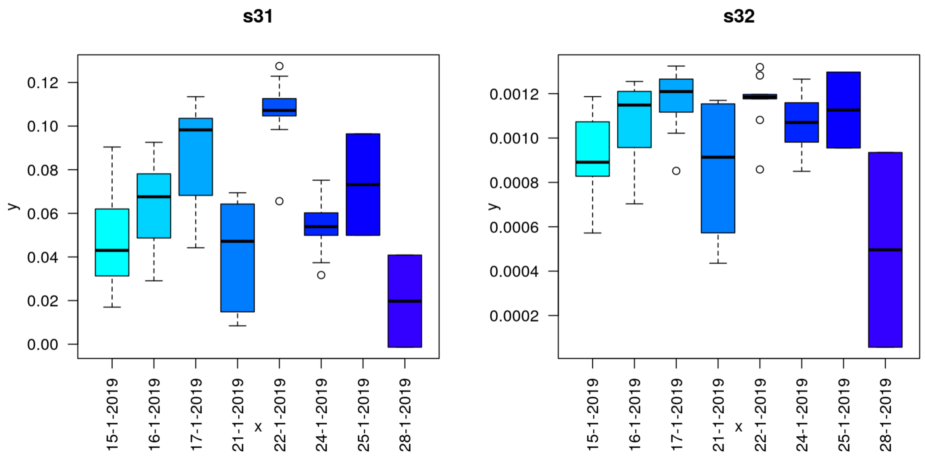

These boxplots show that measurements for cases with gestational age > 27 weeks occur less often for some measurement dates. Indeed, if we examine the number of cases per measurement date this is clear:

```
par(mfrow=c(1, 3), las = 2)
barplot(table(data.ann$Date_of_sample_measurement[sel27]), col = "purple",
        main = "Cases with gest age > 27w")
barplot(table(data.ann$Date_of_sample_measurement), col = "blue",
        main = "All cases")
barplot(table(data.ann$Date_of_sample_measurement[!sel27]), col = "darkgreen",
        main = "Cases with gest age <= 27w")
```

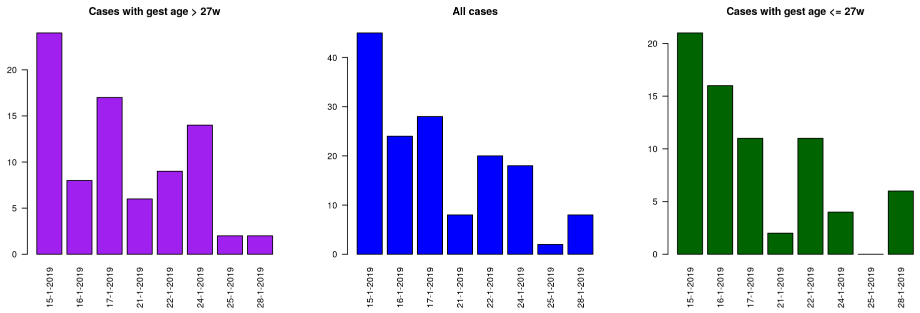

So using this group as reference for fitting the regression may bias results, as the average over just 2 measurements may be an unreliable estimate of the mean for these dates. The best alternative seems to be to center the date according to measurement date, using all cases. Then effects that are linked to a single subset of cases (say, determined according to gestational age) will not be eliminated, as the centering is done using all cases.

Correct for date of measurement

We can correct for the measurement date effect either by fitting a regression model of the sensor data, then using the residuals for further analyses, or by adding the measurement date to the model when studying other effects. The former is more convenient for displaying results via graphs, whilst the latter is more elegant. I will use the former for convenience.

```
norm.sens <- data.sens
for(xj in 1:ncol(data.sens))
{
  norm.sens[, xj] <- residuals(lm(data.sens[, xj] ~ da
ta.ann$Date_of_sample_measurement,
                                  na.action = "na.excl
ude"))
}
sel.nona <- rowSums(is.na(norm.sens)) < ncol(norm.sens
)
```

```
par(las=2, mfrow = c(1, 2))
for(xc in 1:ncol(data.sens))
  plot(data.ann$Date_of_sample_measurement, norm.sens[
, xc],
       col = cols, main = paste(colnames(data.sens)[xc
], "date corr"))
```

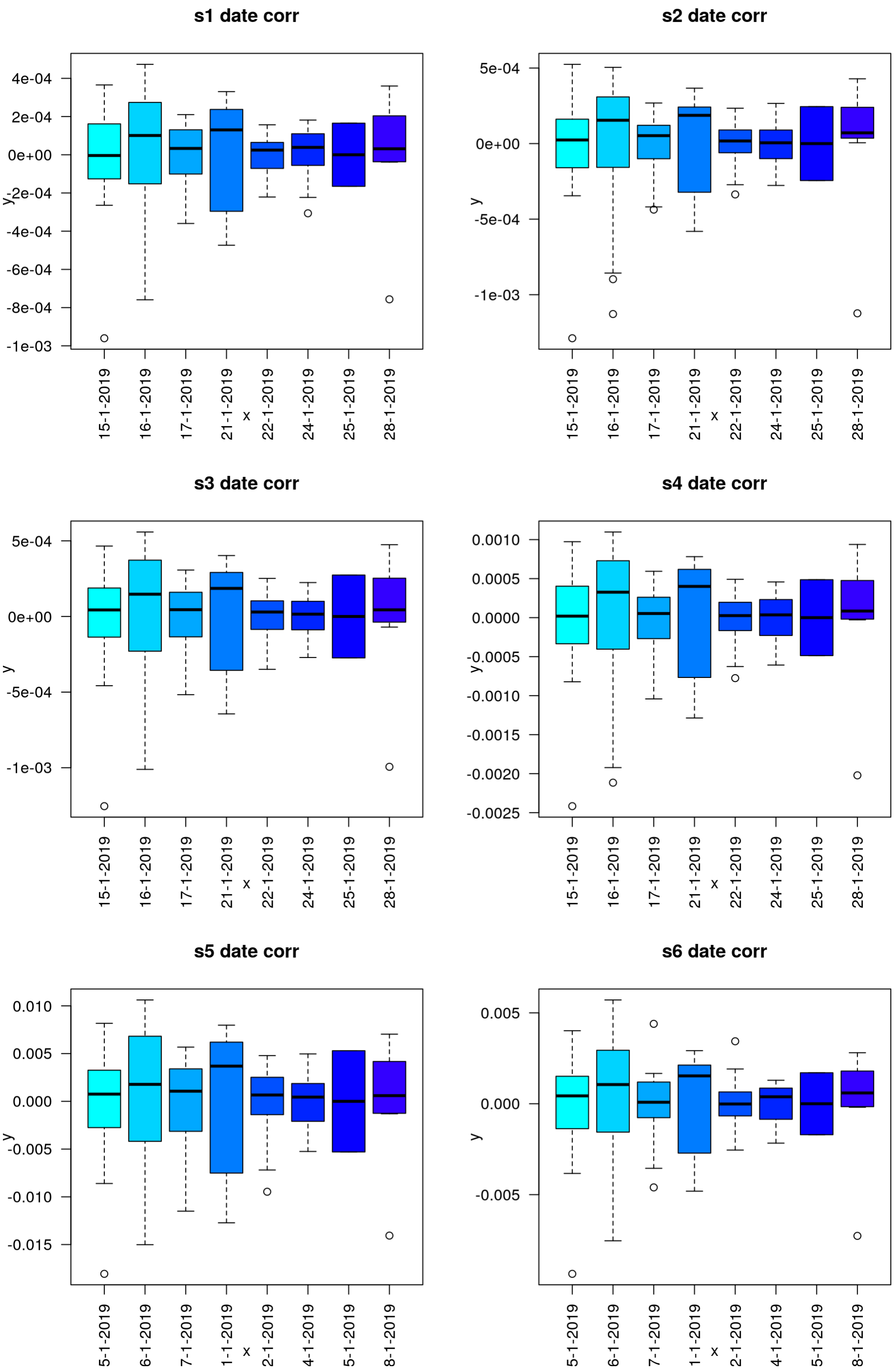
s1 date corr
4e-04
2e-04
0e+00
-2e-04
-4e-04
-6e-04
-8e-04
-1e-03
y
15-1-2019
16-1-2019
17-1-2019
21-1-2019
22-1-2019
24-1-2019
25-1-2019
28-1-2019
x
s2 date corr
5e-04
0e+00
-5e-04
-1e-03
y
15-1-2019
16-1-2019
17-1-2019
21-1-2019
22-1-2019
24-1-2019
25-1-2019
28-1-2019
x
s3 date corr
5e-04
0e+00
-5e-04
-1e-03
y
15-1-2019
16-1-2019
17-1-2019
21-1-2019
22-1-2019
24-1-2019
25-1-2019
28-1-2019
x
s4 date corr
0.0010
0.0005
0.0000
-0.0005
-0.0010
-0.0015
-0.0020
-0.0025
y
15-1-2019
16-1-2019
17-1-2019
21-1-2019
22-1-2019
24-1-2019
25-1-2019
28-1-2019
x
s5 date corr
0.010
0.005
0.000
-0.005
-0.010
-0.015
y
5-1-2019
6-1-2019
7-1-2019
1-1-2019
2-1-2019
4-1-2019
5-1-2019
8-1-2019
x
s6 date corr
0.005
0.000
-0.005
y
5-1-2019
6-1-2019
7-1-2019
1-1-2019
2-1-2019
4-1-2019
5-1-2019
8-1-2019
x

s7 date corr

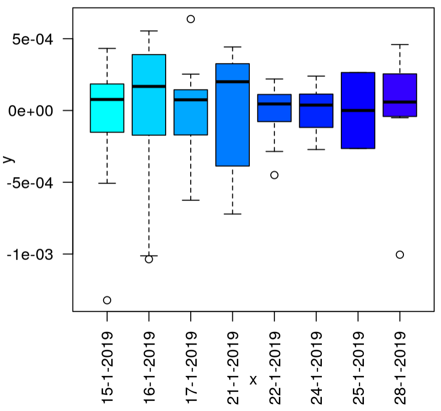

s8 date corr

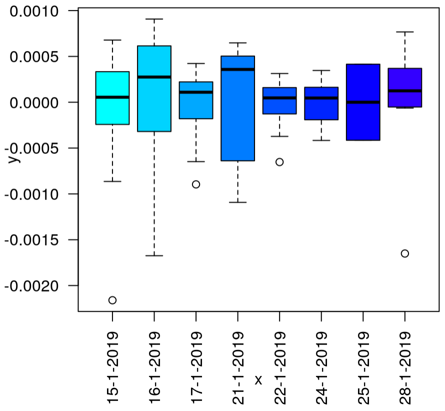

s9 date corr

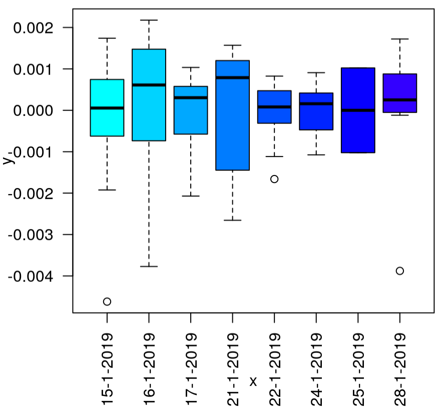

s10 date corr

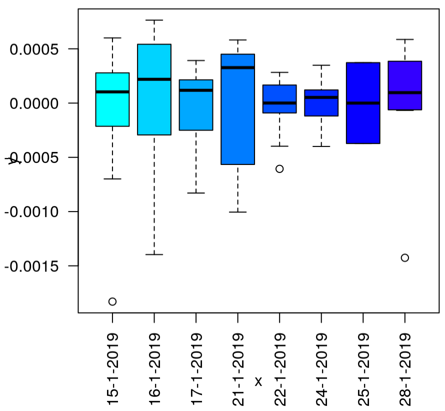

s11 date corr

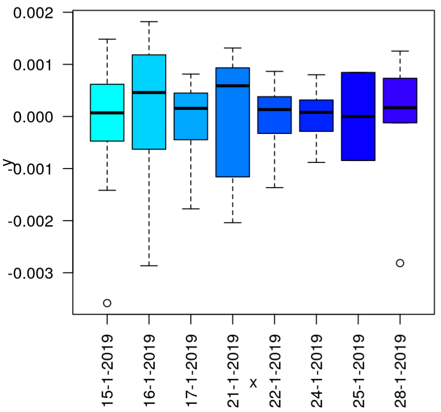

s12 date corr

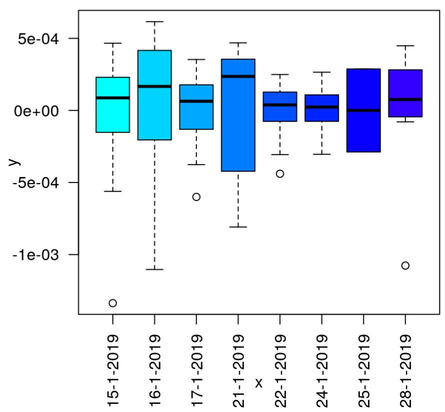

s13 date corr

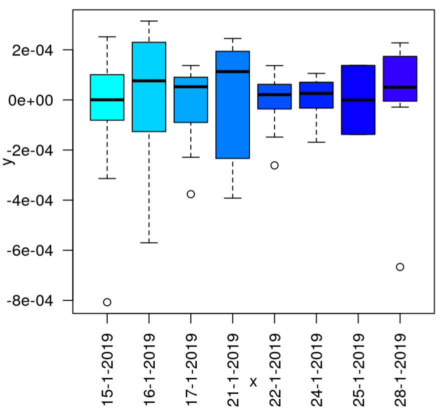

s14 date corr

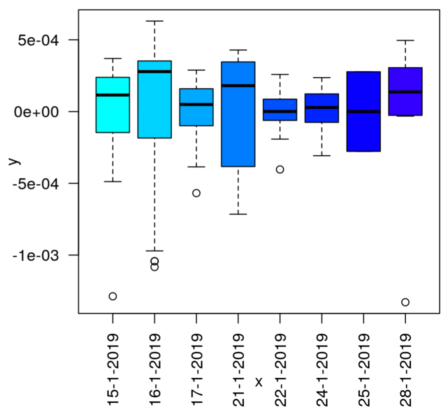

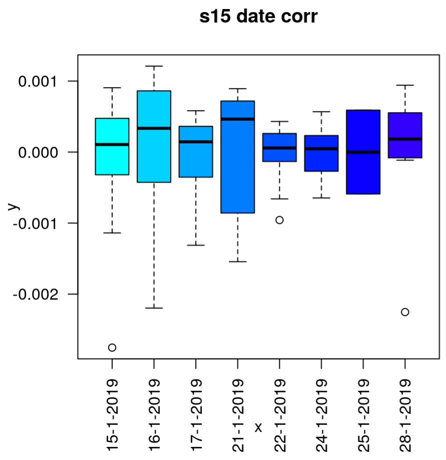
s15 date corr
0.001
0.000
-0.001
-0.002
y
15-1-2019
16-1-2019
17-1-2019
21-1-2019
22-1-2019
24-1-2019
25-1-2019
28-1-2019
x

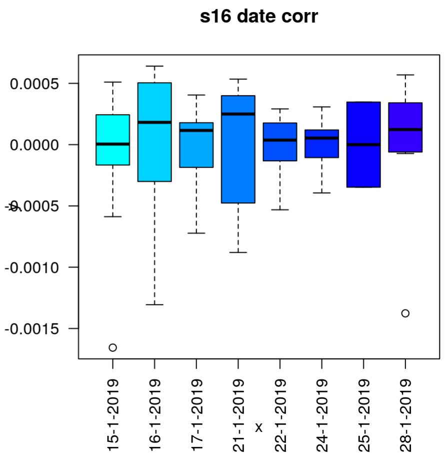
s16 date corr
0.0005
0.0000
-0.0005
-0.0010
-0.0015
y
15-1-2019
16-1-2019
17-1-2019
21-1-2019
22-1-2019
24-1-2019
25-1-2019
28-1-2019
x

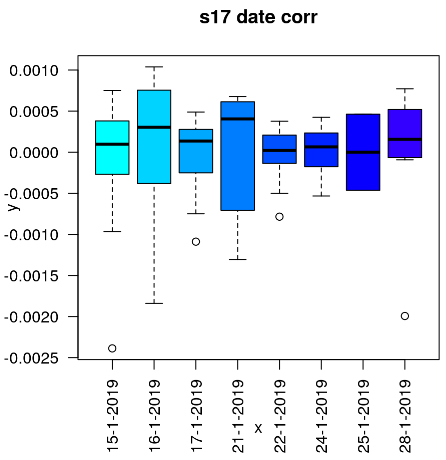
s17 date corr
0.0010
0.0005
0.0000
-0.0005
-0.0010
-0.0015
-0.0020
-0.0025
y
15-1-2019
16-1-2019
17-1-2019
21-1-2019
22-1-2019
24-1-2019
25-1-2019
28-1-2019
x

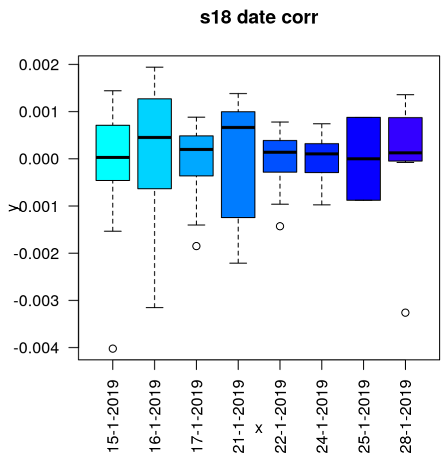
s18 date corr
0.002
0.001
0.000
-0.001
-0.002
-0.003
-0.004
y
15-1-2019
16-1-2019
17-1-2019
21-1-2019
22-1-2019
24-1-2019
25-1-2019
28-1-2019
x

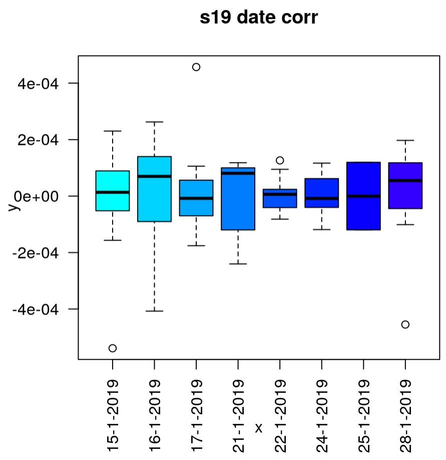
s19 date corr
4e-04
2e-04
0e+00
-2e-04
-4e-04
y
15-1-2019
16-1-2019
17-1-2019
21-1-2019
22-1-2019
24-1-2019
25-1-2019
28-1-2019
x

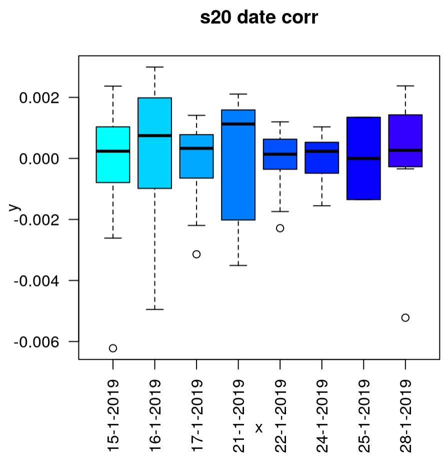
s20 date corr
0.002
0.000
-0.002
-0.004
-0.006
y
15-1-2019
16-1-2019
17-1-2019
21-1-2019
22-1-2019
24-1-2019
25-1-2019
28-1-2019
x

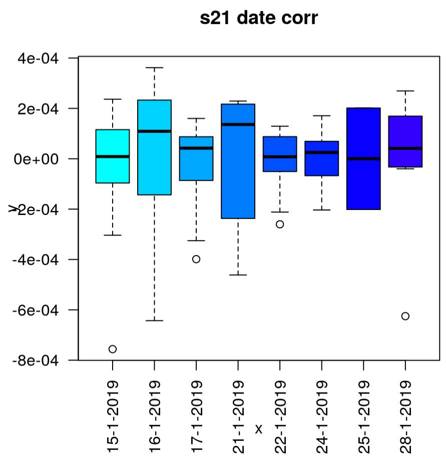
s21 date corr
4e-04
2e-04
0e+00
-2e-04
-4e-04
-6e-04
-8e-04
y
15-1-2019
16-1-2019
17-1-2019
21-1-2019
22-1-2019
24-1-2019
25-1-2019
28-1-2019
x

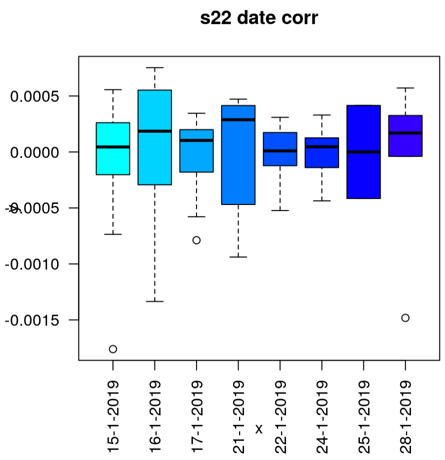
s22 date corr
0.0005
0.0000
-0.0005
-0.0010
-0.0015
y
15-1-2019
16-1-2019
17-1-2019
21-1-2019
22-1-2019
24-1-2019
25-1-2019
28-1-2019
x

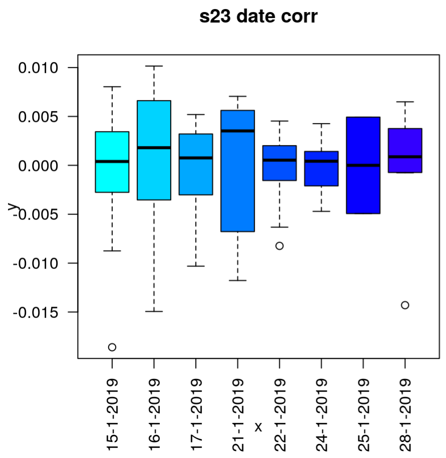
s23 date corr
0.010
0.005
0.000
-0.005
-0.010
-0.015
y
x
15-1-2019
16-1-2019
17-1-2019
21-1-2019
22-1-2019
24-1-2019
25-1-2019
28-1-2019

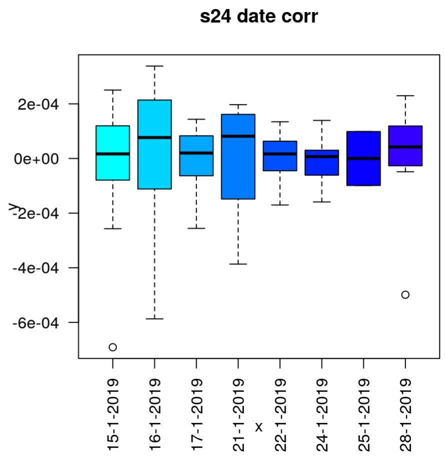
s24 date corr
2e-04
0e+00
-2e-04
-4e-04
-6e-04
y
x
15-1-2019
16-1-2019
17-1-2019
21-1-2019
22-1-2019
24-1-2019
25-1-2019
28-1-2019

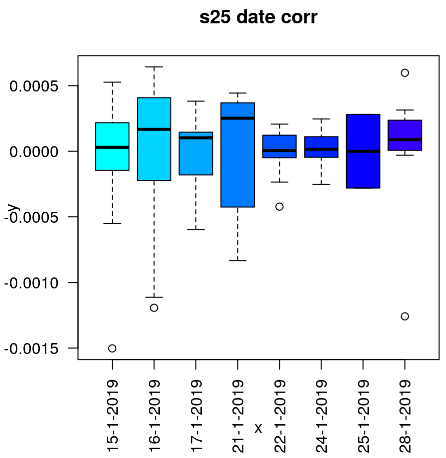
s25 date corr
0.0005
0.0000
-0.0005
-0.0010
-0.0015
y
x
15-1-2019
16-1-2019
17-1-2019
21-1-2019
22-1-2019
24-1-2019
25-1-2019
28-1-2019

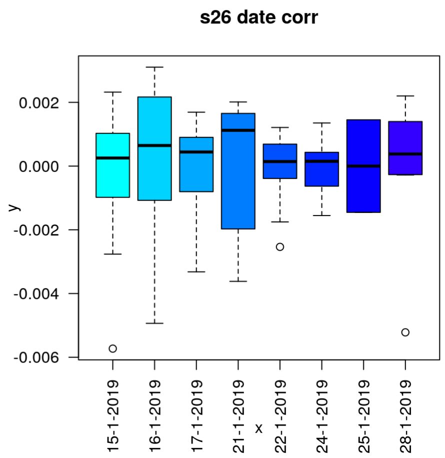
s26 date corr
0.002
0.000
-0.002
-0.004
-0.006
y
x
15-1-2019
16-1-2019
17-1-2019
21-1-2019
22-1-2019
24-1-2019
25-1-2019
28-1-2019

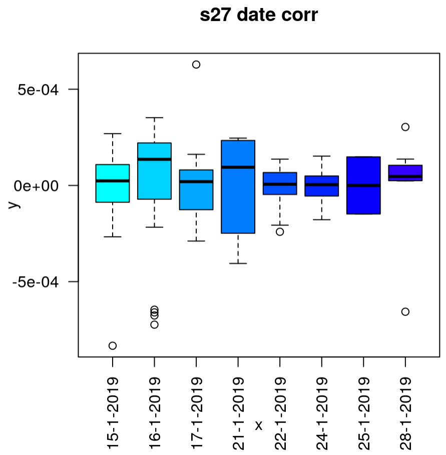
s27 date corr
5e-04
0e+00
-5e-04
y
x
15-1-2019
16-1-2019
17-1-2019
21-1-2019
22-1-2019
24-1-2019
25-1-2019
28-1-2019

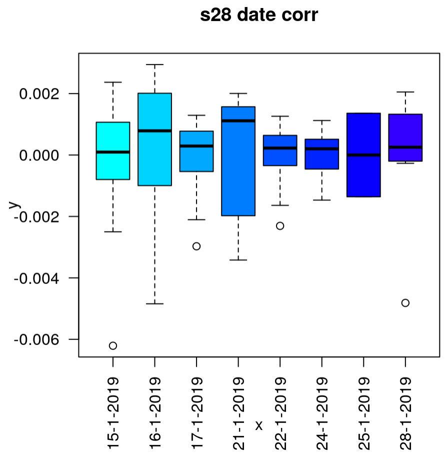
s28 date corr
0.002
0.000
-0.002
-0.004
-0.006
y
x
15-1-2019
16-1-2019
17-1-2019
21-1-2019
22-1-2019
24-1-2019
25-1-2019
28-1-2019

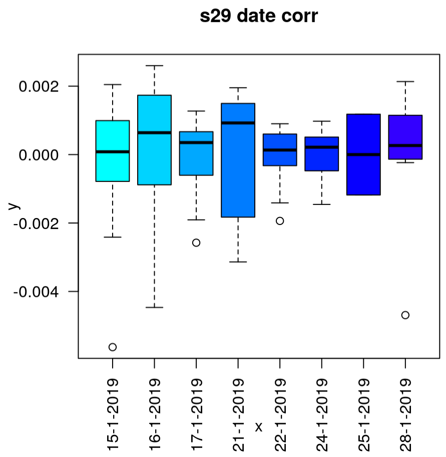
s29 date corr
0.002
0.000
-0.002
-0.004
y
x
15-1-2019
16-1-2019
17-1-2019
21-1-2019
22-1-2019
24-1-2019
25-1-2019
28-1-2019

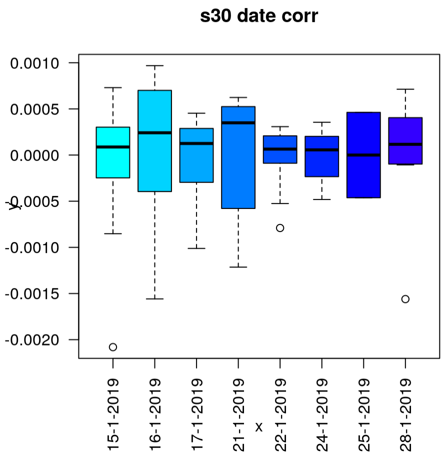
s30 date corr
0.0010
0.0005
0.0000
-0.0005
-0.0010
-0.0015
-0.0020
y
x
15-1-2019
16-1-2019
17-1-2019
21-1-2019
22-1-2019
24-1-2019
25-1-2019
28-1-2019

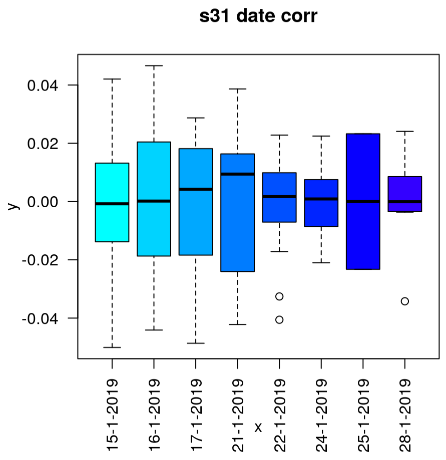
s31 date corr
y
0.04
0.02
0.00
-0.02
-0.04
x
15-1-2019
16-1-2019
17-1-2019
21-1-2019
22-1-2019
24-1-2019
25-1-2019
28-1-2019

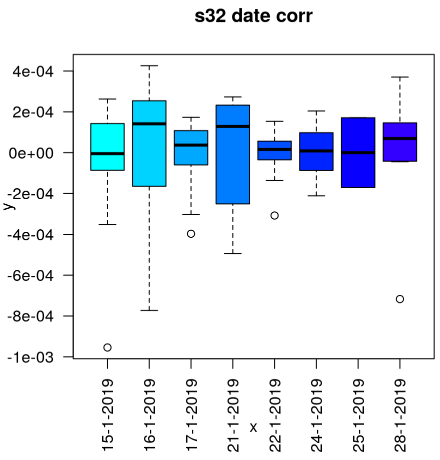
s32 date corr
y
4e-04
2e-04
0e+00
-2e-04
-4e-04
-6e-04
-8e-04
-1e-03
x
15-1-2019
16-1-2019
17-1-2019
21-1-2019
22-1-2019
24-1-2019
25-1-2019
28-1-2019

Sensitivity and gestational age

Then we make comparisons between sensor measurements for different gestational ages. For each comparison, we take one time point only at a time: 7 days after birth, 14 days after birth, 21 days after birth (variable `Day_of_life_measured`). These comparisons are done with the global test first, using all sensors, and subsequently with an F-test from a one-way anova.

```
# Define objects which will contain the results
gt.mat <- matrix(1, nrow = 4, ncol = length(unique(dat
a.ann$Day_of_life_measured)))
rownames(gt.mat) <- c("GesAge", "GesAge after Delivery
", "GesAge after BirthWeight",
                      "GesAge after Feed")
colnames(gt.mat) <- sort(unique(data.ann$Day_of_life_m
easured))
t.mat <- matrix(1, nrow = ncol(norm.sens), ncol = leng
th(unique(data.ann$Day_of_life_measured)))
rownames(t.mat) <- colnames(norm.sens)
colnames(t.mat) <- sort(unique(data.ann$Day_of_life_me
asured))
# Variables of interest:
# f.gesage
# f.deliv
# f.bw
# f.feed
```

```
myday <- 7
myname1 <- paste(myday,"afterBirth", sep="")
selday <- data.ann$Day_of_life_measured == myday
mydata <- norm.sens[selday, ]
f.g1 <- factor(as.character(f.gesage[ selday ]))
f.d1 <- factor(as.character(f.deliv[ selday ]))
f.bw1 <- factor(as.character(f.bw[ selday ]))
f.fe1 <- factor(as.character(f.feed[ selday ]))
#
# For the day of life chosen, run both the global test
for all sensors
# and the Student's-t test per sensor, to explain norm
.sens variability
# by the variables of interest above
#
gt.options(transpose = TRUE)
gt.g <- p.value(gt(f.g1, ~ mydata))
gt.del <- p.value(gt(f.g1, alternative = ~ mydata + f.
d1,  null = ~ f.d1))
gt.bw  <- p.value(gt(f.g1, alternative = ~ mydata + f.
bw1, null = ~ f.bw1))
gt.fe  <- p.value(gt(f.g1, alternative = ~ mydata + f.
fe1, null = ~ f.fe1))
t.pvals <- NULL
for(xj in 1:ncol(mydata)) t.pvals <- c(t.pvals,
                                       anova(lm(mydata
[, xj] ~ f.g1))[ "Pr(>F)"][1,1])

### Storing results
t.mat[, colnames(t.mat) == myday] <- t.pvals
gt.mat[, colnames(gt.mat) == myday] <- c(gt.g, gt.del,
gt.bw, gt.fe)
```

```
myday <- 14
myname1 <- paste(myday,"afterBirth", sep="")
selday <- data.ann$Day_of_life_measured == myday
mydata <- norm.sens[selday, ]
f.g1 <- factor(as.character(f.gesage[ selday ]))
f.d1 <- factor(as.character(f.deliv[ selday ]))
f.bw1 <- factor(as.character(f.bw[ selday ]))
f.fe1 <- factor(as.character(f.feed[ selday ]))
#
# For the day of life chosen, run both the global test
for all sensors
# and the Student's-t test per sensor, to explain norm
.sens variability
# by the variables of interest above
#
gt.options(transpose = TRUE)
gt.g <- p.value(gt(f.g1, ~ mydata))
gt.del <- p.value(gt(f.g1, alternative = ~ mydata + f.
d1,  null = ~ f.d1))
gt.bw  <- p.value(gt(f.g1, alternative = ~ mydata + f.
bw1, null = ~ f.bw1))
gt.fe  <- p.value(gt(f.g1, alternative = ~ mydata + f.
fe1, null = ~ f.fe1))
t.pvals <- NULL
for(xj in 1:ncol(mydata)) t.pvals <- c(t.pvals,
                                       anova(lm(mydata
[, xj] ~ f.g1))[ "Pr(>F)"][1,1])

### Storing results
t.mat[, colnames(t.mat) == myday] <- t.pvals
gt.mat[, colnames(gt.mat) == myday] <- c(gt.g, gt.del,
gt.bw, gt.fe)
```

```
myday <- 21
myname1 <- paste(myday,"afterBirth", sep="")
selday <- data.ann$Day_of_life_measured == myday
mydata <- norm.sens[selday, ]
f.g1 <- factor(as.character(f.gesage[ selday ]))
f.d1 <- factor(as.character(f.deliv[ selday ]))
f.bw1 <- factor(as.character(f.bw[ selday ]))
f.fe1 <- factor(as.character(f.feed[ selday ]))
#
# For the day of life chosen, run both the global test
for all sensors
# and the Student's-t test per sensor, to explain norm
.sens variability
# by the variables of interest above
#
gt.options(transpose = TRUE)
gt.g <- p.value(gt(f.g1, ~ mydata))
gt.del <- p.value(gt(f.g1, alternative = ~ mydata + f.
d1,  null = ~ f.d1))
gt.bw  <- p.value(gt(f.g1, alternative = ~ mydata + f.
bw1, null = ~ f.bw1))
gt.fe  <- p.value(gt(f.g1[!is.na(f.fe1)],
                     alternative = ~ mydata[!is.na(f.f
e1), ] + f.fe1[!is.na(f.fe1)],
                     null = ~ f.fe1[!is.na(f.fe1)]))
t.pvals <- NULL
for(xj in 1:ncol(mydata)) t.pvals <- c(t.pvals,
                                       anova(lm(mydata
[, xj] ~ f.g1))[ "Pr(>F)"][1,1])

### Storing results
t.mat[, colnames(t.mat) == myday] <- t.pvals
gt.mat[, colnames(gt.mat) == myday] <- c(gt.g, gt.del,
gt.bw, gt.fe)
```

ANOVA results can be summarized in various ways. For example, we look at the smallest p-value per day, across all sensors:

```
round(apply(t.mat, 2, min), 3)
```

```
##     7    14    21
## 0.358 0.131 0.609
```

This already indicates that there is no effect of gestational age.

We also run a global test, which explains gestational age using all sensors at once, first with only the sensor data, then checking the added value of the sensor data after correcting for delivery, birth weight and feed, one at a time. The p-values obtained are:

```
round(gt.mat, 3)
```

```
##                               7    14    21
## GesAge                    0.383 0.656 0.955
## GesAge after Delivery     0.422 0.667 0.973
## GesAge after BirthWeight  0.321 0.871 0.861
## GesAge after Feed         0.640 0.472 0.790
```

This confirms the results from ANOVA.

Mode of delivery and sensor data

We also looked at the effect of mode of delivery on sensor measurements made at 7 days after birth, 14 or 21.

```
# Define objects which will contain the results
gt.mat <- matrix(1, nrow = 1, ncol = length(unique(dat
a.ann$Day_of_life_measured)))
rownames(gt.mat) <- c("Delivery")
colnames(gt.mat) <- sort(unique(data.ann$Day_of_life_m
easured))
t.mat <- matrix(1, nrow = ncol(norm.sens), ncol = leng
th(unique(data.ann$Day_of_life_measured)))
rownames(t.mat) <- colnames(norm.sens)
colnames(t.mat) <- sort(unique(data.ann$Day_of_life_me
asured))
# Variables of interest:
# f.deliv
```

```
myday <- 7
myname1 <- paste(myday,"afterBirth", sep="")
selday <- data.ann$Day_of_life_measured == myday
mydata <- norm.sens[selday, ]
f.d1 <- factor(as.character(f.deliv[ selday ]))
#
# For the day of life chosen, run both the global test
for all sensors
# and the Student's-t test per sensor, to explain norm
.sens variability
# by the variables of interest above
#
gt.options(transpose = TRUE)
gt.g <- p.value(gt(f.d1, ~ mydata))
t.pvals <- NULL
for(xj in 1:ncol(mydata)) t.pvals <- c(t.pvals,
                                       anova(lm(mydata
[, xj] ~ f.d1))[ "Pr(>F)"][1,1])

### Storing results
t.mat[, colnames(t.mat) == myday] <- t.pvals
gt.mat[, colnames(gt.mat) == myday] <- gt.g
```

```
myday <- 14
myname1 <- paste(myday,"afterBirth", sep="")
selday <- data.ann$Day_of_life_measured == myday
mydata <- norm.sens[selday, ]
f.d1 <- factor(as.character(f.deliv[ selday ]))
#
# For the day of life chosen, run both the global test
for all sensors
# and the Student's-t test per sensor, to explain norm
.sens variability
# by the variables of interest above
#
gt.options(transpose = TRUE)
gt.g <- p.value(gt(f.d1, ~ mydata))
t.pvals <- NULL
for(xj in 1:ncol(mydata)) t.pvals <- c(t.pvals,
                                       anova(lm(mydata
[, xj] ~ f.d1))[ "Pr(>F)"][1,1])

### Storing results
t.mat[, colnames(t.mat) == myday] <- t.pvals
gt.mat[, colnames(gt.mat) == myday] <- gt.g
```

```r
myday <- 21
myname1 <- paste(myday,"afterBirth", sep="")
selday <- data.ann$Day_of_life_measured == myday
mydata <- norm.sens[selday, ]
f.d1 <- factor(as.character(f.deliv[ selday ]))
#
# For the day of life chosen, run both the global test
for all sensors
# and the Student's-t test per sensor, to explain norm
.sens variability
# by the variables of interest above
#
gt.options(transpose = TRUE)
gt.g <- p.value(gt(f.d1, ~ mydata))
t.pvals <- NULL
for(xj in 1:ncol(mydata)) t.pvals <- c(t.pvals,
                                       anova(lm(mydata
[, xj] ~ f.d1))[ "Pr(>F)"][1,1])

### Storing results
t.mat[, colnames(t.mat) == myday] <- t.pvals
gt.mat[, colnames(gt.mat) == myday] <- gt.g
```

ANOVA results can be summarized in various ways. For example, we look at the smallest p-value per day, across all sensors:

```r
round(apply(t.mat, 2, min), 3)
```

```
##     7    14    21
## 0.520 0.503 0.268
```

This already indicates that there is no effect of gestational age.

We also run a global test, which explains gestational age using all sensors at once, first with only the sensor data, then checking the added value of the sensor data after correcting for delivery, birth weight and feed, one at a time. The p-values obtained are:

```r
round(gt.mat, 3)
```

```
##              7    14    21
## Delivery 0.716 0.946 0.331
```

So, we also found no evidence that delivery can display association with the sensor data.

Heatmaps normalized data

For completeness, we make heatmaps of the normalized sensor data.

```
myday <- 7
myname1 <- paste(myday,"afterBirth", sep="")
data.ann$Feeding_mode_prior_day_of_life_analyzed[ data
.ann$Feeding_mode_prior_day_of_life_analyzed < 0] <- N
A
selday <- data.ann$Day_of_life_measured == myday
mydata <- norm.sens[selday & sel.nona, ]
d.ann <- data.ann[selday & sel.nona, ]
f.gesage <- factor(cut(d.ann$Gestational_age_days, bre
aks = c(0, 27*7, 30*7 )),
                   labels = c("<= 27w", "> 27w"))
f.deliv <- factor(d.ann$Mode_of_delivery, labels = c("
vaginal", "c-section"))
```

```
myvar <- var.in.colour(f.gesage, mystart=0.4, myend=0.
7)
heatmap.2(mydata, trace = 'none', col = 'bluered', #br
eaks = seq(0, 0.01, by =0.001),
          RowSideColors = myvar[[1]], main = paste("We
ek", myday))
legend("topright", legend = myvar[[3]], fill = myvar[[
2]], cex=0.5)
```

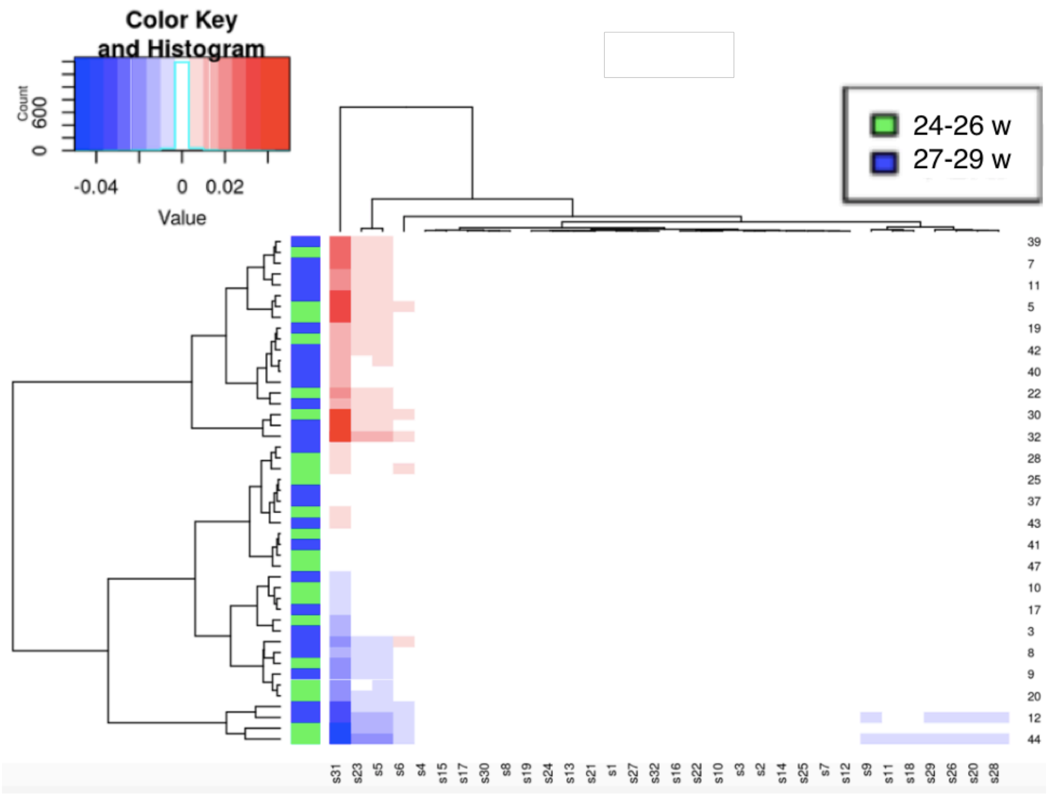

Figure S1. *Heatmap for corrected sensor data by gestational age at day 7 of life.* Sensor outcomes are equally distributed between groups with most centered data close to 0 (white color). (GA ≤ 27 weeks: green, > 27 weeks: blue color).

```
myvar <- var.in.colour(f.deliv, mystart=0.4, myend=0.7
)
heatmap.2(mydata, trace = 'none', col = 'bluered', #br
eaks = seq(0, 0.01, by =0.001),
          RowSideColors = myvar[[1]], main = paste("We
ek", myday))
legend("topright", legend = myvar[[3]], fill = myvar[[
2]], cex=0.5)
```

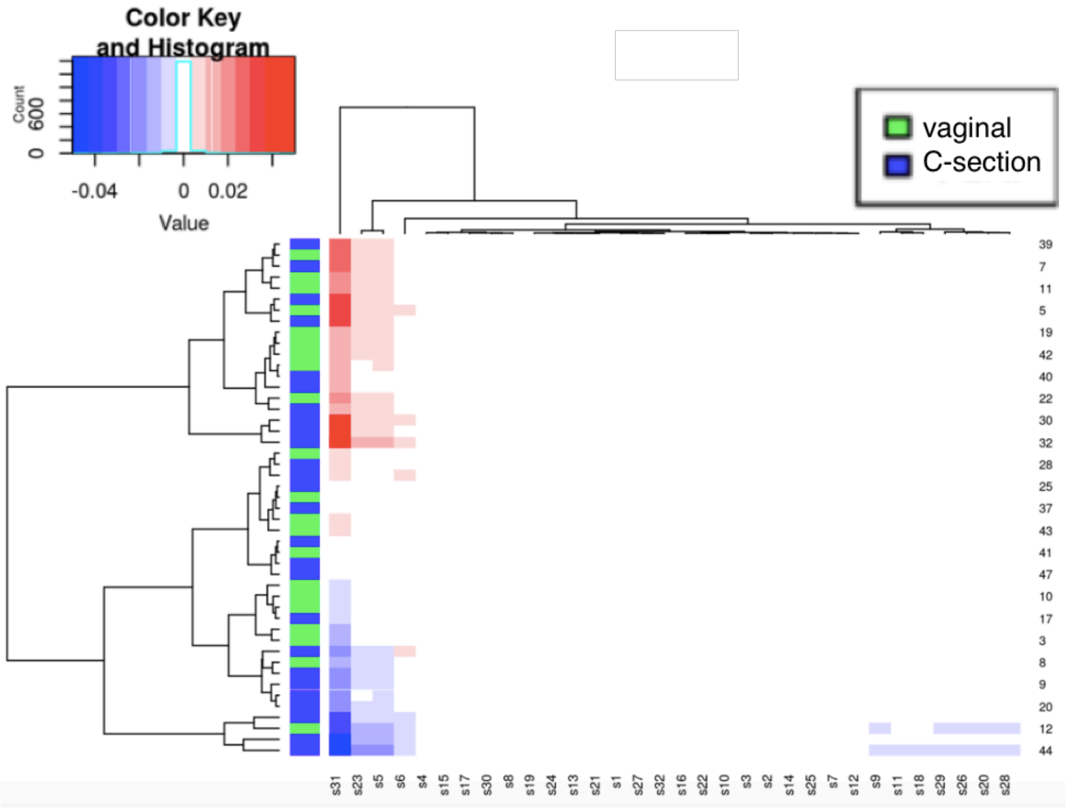

Figure S2. *Heatmap for corrected sensor data by mode of delivery at day 7 of life.* Sensor outcomes are equally distributed between groups with most centered data close to 0 (white color). (vaginal delivery: green, C-section: blue color).

```
myday <- 14
myname1 <- paste(myday,"afterBirth", sep="")
selday <- data.ann$Day_of_life_measured == myday
mydata <- norm.sens[selday & sel.nona, ]
d.ann <- data.ann[selday & sel.nona, ]
f.gesage <- factor(cut(d.ann$Gestational_age_days, bre
aks = c(0, 27*7, 30*7 )),
                   labels = c("<= 27w", "> 27w"))
f.deliv <- factor(d.ann$Mode_of_delivery, labels = c("
vaginal", "c-section"))
f.bw <- factor(cut(d.ann$Birth.weight_grams, breaks =
c(0, 900, 1200, 2000)),
               labels = c("<= 900g", "900g < x <= 1200
g","> 1200g"))
data.ann$Feeding_mode_prior_day_of_life_analyzed[ data
.ann$Feeding_mode_prior_day_of_life_analyzed < 0] <- N
A
myvar <- var.in.colour(f.gesage, mystart=0.4, myend=0.
7)
heatmap.2(mydata, trace = 'none', col = 'bluered', #br
eaks = seq(0, 0.01, by =0.001),
          RowSideColors = myvar[[1]], main = paste("We
ek", myday))
legend("topright", legend = myvar[[3]], fill = myvar[[
2]], cex=0.5)
```

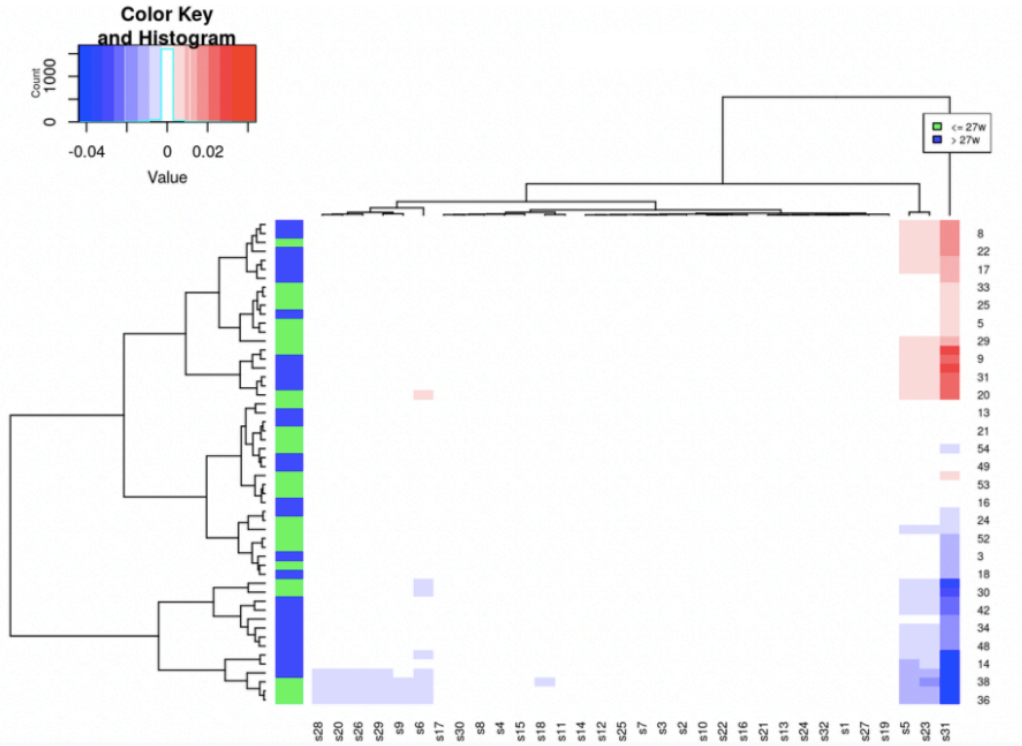

Figure S3. *Heatmap for corrected sensor data by gestational age at day 14 of life.* Sensor outcomes are equally distributed between groups with most centered data close to 0 (white color). (GA ≤ 27 weeks: green, > 27 weeks: blue color).

```
myvar <- var.in.colour(f.deliv, mystart=0.4, myend=0.7
)
heatmap.2(mydata, trace = 'none', col = 'bluered', #br
eaks = seq(0, 0.01, by =0.001),
          RowSideColors = myvar[[1]], main = paste("We
ek", myday))
legend("topright", legend = myvar[[3]], fill = myvar[[
2]], cex=0.5)
```

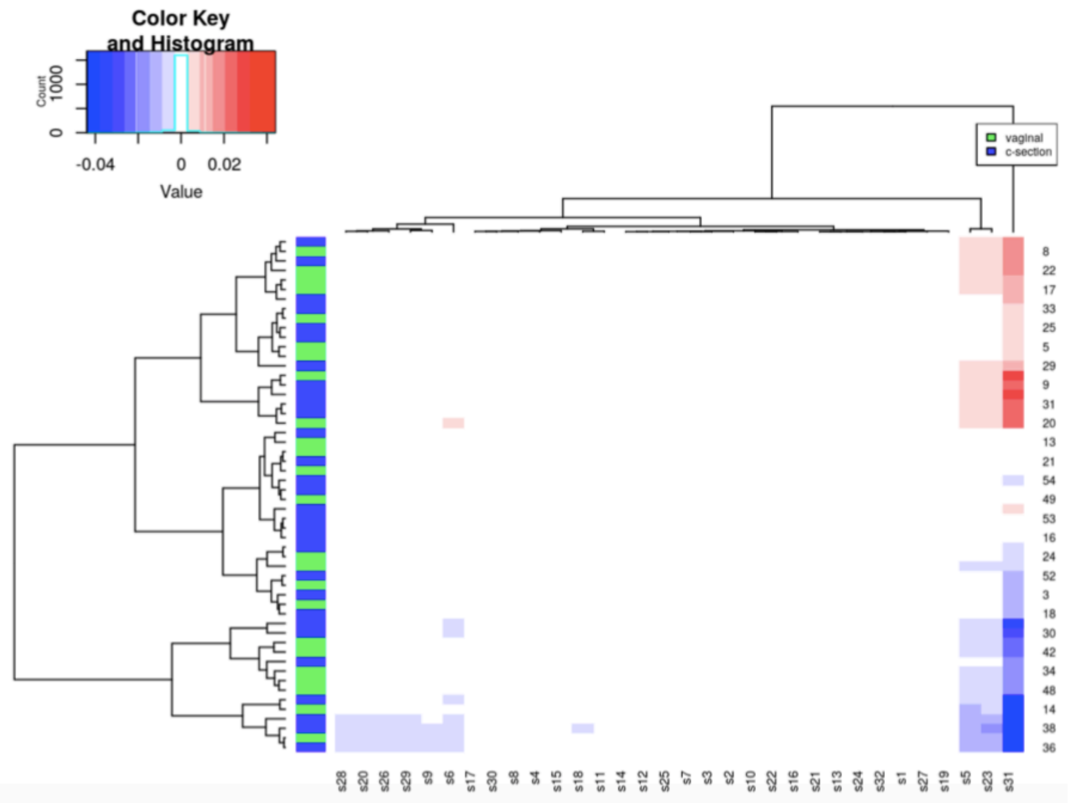

Figure S4. *Heatmap for corrected sensor data by mode of delivery at day 14 of life.* Sensor outcomes are equally distributed between groups with most centered data close to 0 (white color). (GA ≤ 27 weeks: green, > 27 weeks: blue color).

```
myday <- 21
myname1 <- paste(myday,"afterBirth", sep="")
selday <- data.ann$Day_of_life_measured == myday
mydata <- norm.sens[selday & sel.nona, ]
d.ann <- data.ann[selday & sel.nona, ]
f.gesage <- factor(cut(d.ann$Gestational_age_days, bre
aks = c(0, 27*7, 30*7 )),
                   labels = c("<= 27w", "> 27w"))
f.deliv <- factor(d.ann$Mode_of_delivery, labels = c("
vaginal", "c-section"))
myvar <- var.in.colour(f.gesage, mystart=0.4, myend=0.
7)
heatmap.2(mydata, trace = 'none', col = 'bluered', #br
eaks = seq(0, 0.01, by =0.001),
          RowSideColors = myvar[[1]], main = paste("We
ek", myday))
legend("topright", legend = myvar[[3]], fill = myvar[[
2]], cex=0.5)
```

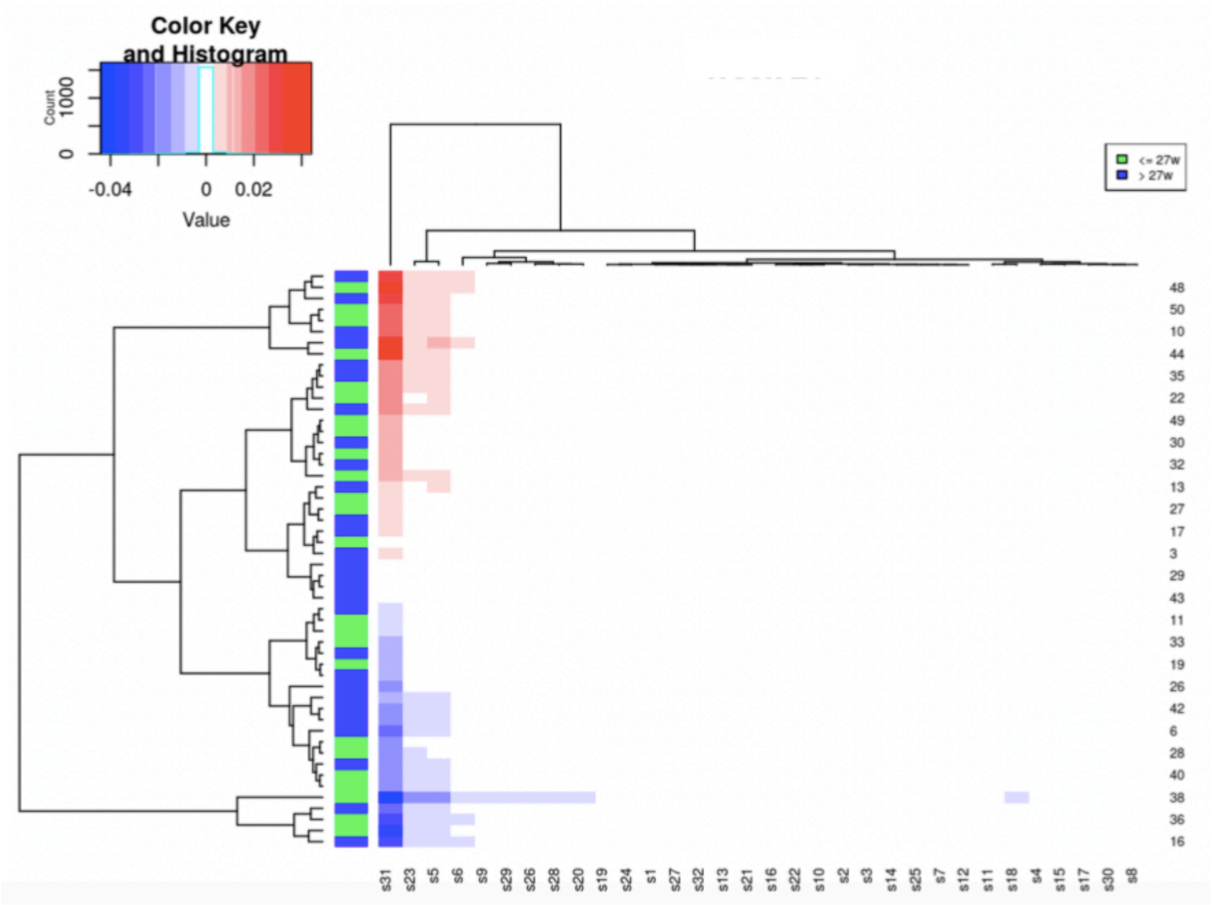

Figure S5. *Heatmap for corrected sensor data by gestational age at day 21 of life.* Sensor outcomes are equally distributed between groups with most centered data close to 0 (white color). (GA ≤ 27 weeks: green, > 27 weeks: blue color).

```
myvar <- var.in.colour(f.deliv, mystart=0.4, myend=0.7
)
heatmap.2(mydata, trace = 'none', col = 'bluered', #br
eaks = seq(0, 0.01, by =0.001),
          RowSideColors = myvar[[1]], main = paste("We
ek", myday))
legend("topright", legend = myvar[[3]], fill = myvar[[
2]], cex=0.5)
```

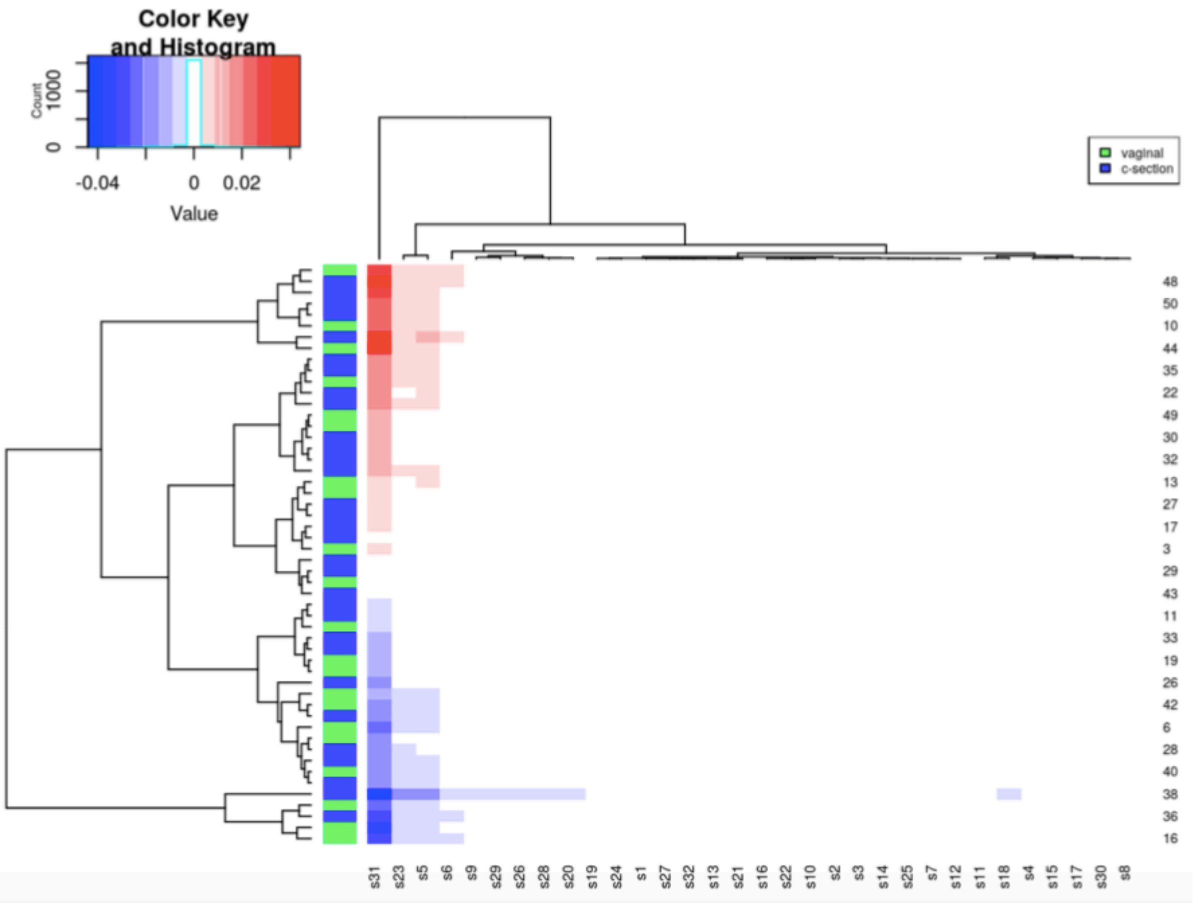

Figure S6. *Heatmap for corrected sensor data by mode of delivery at day 21 of life.* Sensor outcomes are equally distributed between groups with most centered data close to 0 (white color). (GA ≤ 27 weeks: green, > 27 weeks: blue color).

Packages and versions

```
sessionInfo()
```

```
## R version 3.6.3 (2020-02-29)
## Platform: x86_64-pc-linux-gnu (64-bit)
## Running under: Ubuntu 18.04.4 LTS
##
## Matrix products: default
## BLAS:   /usr/lib/x86_64-linux-gnu/blas/libblas.so.3.7.1
## LAPACK: /usr/lib/x86_64-linux-gnu/lapack/liblapack.so.3.7.1
##
## locale:
##  [1] LC_CTYPE=en_US.UTF-8       LC_NUMERIC=C
##  [3] LC_TIME=nl_NL.UTF-8        LC_COLLATE=en_US.UTF-8
##  [5] LC_MONETARY=nl_NL.UTF-8    LC_MESSAGES=en_US.UTF-8
##  [7] LC_PAPER=nl_NL.UTF-8       LC_NAME=C
##  [9] LC_ADDRESS=C               LC_TELEPHONE=C
## [11] LC_MEASUREMENT=nl_NL.UTF-8 LC_IDENTIFICATION=C
##
## attached base packages:
## [1] stats     graphics  grDevices utils     datasets  methods   base
##
## other attached packages:
## [1] globaltest_5.38.0 survival_3.1-11   gplots_3.0.1.1
##
## loaded via a namespace (and not attached):
##  [1] Rcpp_1.0.3           pillar_1.4.2         compiler_3.6.3
##  [4] bitops_1.0-6         tools_3.6.3          zeallot_0.1.0
##  [7] digest_0.6.23        bit_1.1-14           memoise_1.1.0
## [10] tibble_2.1.3         annotate_1.62.0      evaluate_0.14
## [13] RSQLite_2.1.2        lattice_0.20-40      pkgconfig_2.0.3
## [16] rlang_0.4.2          Matrix_1.2-18        DBI_1.0.0
## [19] yaml_2.2.0           parallel_3.6.3       xfun_0.11
## [22] stringr_1.4.0        knitr_1.26           IRanges_2.18.3
## [25] S4Vectors_0.22.1     vctrs_0.2.0          gtools_3.8.1
## [28] caTools_1.17.1.2     stats4_3.6.3         bit64_0.9-7
## [31] grid_3.6.3           Biobase_2.44.0       AnnotationDbi_1.46.1
## [34] XML_3.98-1.20        rmarkdown_1.18       gdata_2.18.0
## [37] blob_1.2.0           magrittr_1.5         backports_1.1.5
## [40] htmltools_0.4.0      splines_3.6.3        BiocGenerics_0.30.0
## [43] xtable_1.8-4         KernSmooth_2.23-16   stringi_1.4.3
## [46] RCurl_1.95-4.12      crayon_1.3.4
```
